# Exploring the potential of end-capping acceptor engineering on indolo[3,2-*b*]indole-based small molecules for efficient organic and perovskite solar cells†

**DOI:** 10.1039/d3ra08639a

**Published:** 2024-02-09

**Authors:** Waqar Ali Zahid, Muhammad Fiaz Ahmad, Waqas Akram, Rabia Iftikhar, Sarah A. Alsalhi, Shaimaa A. M. Abdelmohsen, Javed Iqbal

**Affiliations:** a Department of Chemistry, University of Agriculture Faisalabad 38000 Pakistan javedkhattak79@gmail.com Javed.iqbal@uaf.edu.pk; b Department of Chemistry, University of Education Lahore Pakistan; c Department of Physics, College of Science, Princess Nourah bint Abdulrahman University P.O. Box 84428 Riyadh 11671 Saudi Arabia

## Abstract

Photovoltaic (PV) materials, especially organic and perovskite solar cells are effective candidates for meeting the rising global energy demand. Herein, we have designed indolo[3,2-*b*]indole-based six molecules (IDF1–IDF6) as hole-transporting materials (HTMs) for perovskite solar cells (PSCs) and donor materials for organic solar cells (OSCs). The results demonstrated that IDF1–IDF6 molecules have tight π–π stacking, more negative HOMO levels (−5.50 to −5.31 eV), low bandgaps (1.91 to 2.41 eV), high absorption coefficients, large Stokes shifts, high open-circuit photovoltages (1.31 to 1.50 V), and superior solubility with comparable stability compared with the reference (IDFR) and Spiro-OMeTAD molecules. The high light-harvesting efficiency and low exciton binding energy indicated that IDF1–IDF6 molecules have a higher photocurrent flow ability. The electronic excitation analyses of studied molecules showed that the IDF1–IDF6 molecules show stronger exciton dissociation, low charge coupling, and high intrinsic charge transfer with sharper charge flow than IDFR and Spiro-OMeTAD. Moreover, the high hole hopping rate, high total amount of charge transfer, and low reorganization energy with comparable charge transfer integral demonstrated that the designed molecules have effective hole transport ability for solar cells. Our remarkable results demonstrated that IDF1–IDF6 are advantageous molecules for the manufacturing of efficient PSCs and OSCs, and may have future commercial applications in the solar industry.

## Abstract 2

Photovoltaic (PV) materials, especially organic and perovskite solar cells are effective candidates for meeting the rising global energy demand.

## Graphical abstract

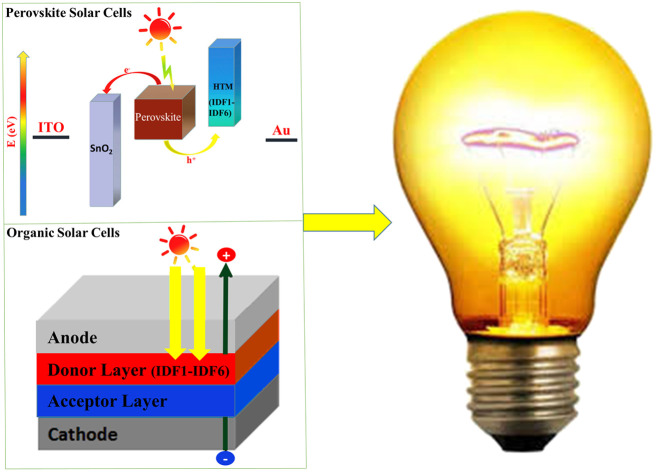

## 1. Introduction

The excessive use of energy resources, the rising global energy demand, and the subsequent environmental crisis, such as the increased consumption of fossil fuels, are issues that need to be addressed immediately and suggest the need for alternative renewable energy sources to avoid further climate change impact.^1,2^ In this regard, photovoltaic (PV) materials, especially organic and perovskite solar cells are effective candidates for aiding this task.^3^ Perovskite solar cells (PSCs) have ideal properties *i.e.*, easy fabrication, band gap tuning, high absorption coefficient, cost-effectiveness, long charge carrier diffusion length, high dielectric constant, highly crystalline structure, low binding energy, and high carrier mobility.^4–8^ On the other hand, organic solar cells (OSCs) have distinctive properties including lightweight, mechanically flexible, highly versatile, low-cost, and solution-based fabrication.^9–14^ The power conversion efficiency (PCE) has increased to around 20% for OSCs and above 25% for PSCs, making them ideal for commercial applications.^15–18^ In OSC configuration, the active layer consists of a p-type organic semiconductor (electron donor) and n-type organic semiconductor (electron acceptor). At present, most OSCs use a polymer donor with a non-fullerene small molecule acceptor, which has significantly improved OSC performance.^19^ The PSCs consist of perovskite material (PM), hole-transporting material (HTM), and electron-transporting material (ETM) in which the PM absorbs solar radiations, resulting in the production of electrons and holes which are transported by the electron-transporting layer, and a hole-transporting layer before being collected by the electrodes.^20^ The poor active layer structure is one of OSCs' limitations. Due to their high crystallinity and strong intermolecular interactions, typical small molecule donors and polymer acceptors are not matched.^21,22^ The low device efficiency in OSC is caused by the blends' frequent large-size phase separation when they interact with polymer acceptors, which restricts exciton diffusion and dissociation.^23,24^

In PSCs, the matched energy levels across the perovskite material, electron-transporting material, and hole-transporting material are essential for facilitating electron and hole extraction at the interface, improving PSCs' photovoltaic properties.^25,26^ HTM plays a key role in perovskite solar cells by facilitating hole conduction, blocking electrons, and shielding the absorber from moisture and metal diffusion from the electrode. The Spiro-OMeTAD is still primary standard HTM utilized in high-performance PSCs, although it is expensive to synthesize and unstable.^27,28^ Hence, the major obstacle to enhancing the photovoltaic properties and efficiency of OSCs and PSCs is the lack of donor and hole-transporting materials. Recently S. H. Hong *et al.* reported indole[3,2-*b*]indole-based HTM (termed DEG-IDIDF) with a PCE of 16.16%.^29^ DEG-IDIDF molecule showed deeper HOMO energy (−5.13 eV), superior hole mobilities (5.91 × 10^−4^ cm^2^ V^−1^ s^−1^), high absorption (440 nm), and short-circuit current density (21.23 mA cm^−2^) with 0.75% fill factor. The photovoltaic properties of the DEG-IDIDF molecule can be further enhanced by introducing the acceptors *via* the thiophene bridge in replacing the alkyl group.

**Fig. 1:**
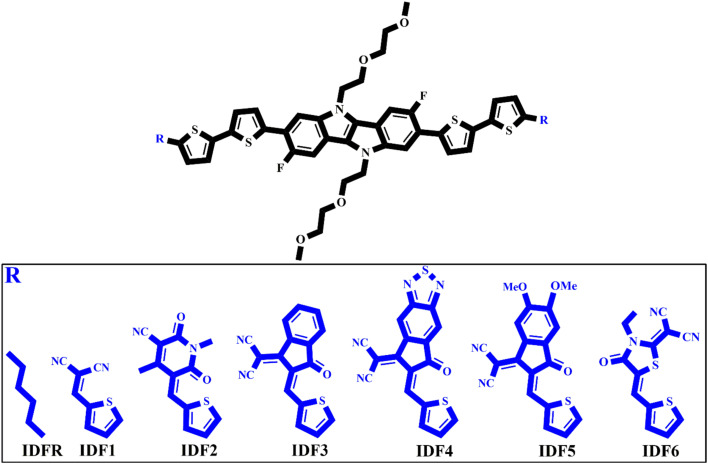
Design schematic strategy for the engineering IDF1, IDF2, IDF3, IDF4, IDF5, and IDF6 molecules derived from reference (IDFR).

Herein, six new molecules namely IDF1, IDF2, IDF3, IDF4, IDF5, and IDF6 are designed which are based on the fluorinated indolo[3,2-*b*]indole-based core with diethylene glycol chains, terthiophene-based side arms, and end-capped cyano- (IDF1), pyridine- (IDF2), indan- (IDF3), thiodiamine indan- (IDF4), methoxy indan- (IDF5), and thiazolidin- (IDF6) based acceptors as presented in Fig. 1. The electronic, solubility, stability, optical, and charge-transporting properties of engineered molecules have been studied *via* density functional theory (DFT) and time-dependent DFT (TD-DFT) approaches and their results are compared with the reference (IDFR) and Spiro-OMeTAD molecules. Our studies focus on the incorporation of acceptors *via* thiophene on the efficiency of designed molecules and can provide some guidelines for the fabrication of highly effective HTMs for PSCs and donor materials for OSCs.

## 2. Computational methodology

The DFT-based computations^30,31^ of the studied molecules are performed in the Gaussian 09W program^32^ and findings are displayed in the GaussView 6.0 software.^33^ To find an appropriate functional, the reference molecule (IDFR) is optimized by using the DFT functionals (ωB97XD, MPW1PW91, CAM-B3LYP, and B3LYP) with the basis set “6-31G (d, p)”. The HOMO and LUMO energies (in eV) of the reference molecule (IDFR) with DFT functionals are −6.66 and −0.08 (for ωB97XD), −4.96 and −1.68 (for MPW1PW91), −5.93 and −0.63 (for CAM-B3LYP), −4.67 and −1.76 (for B3LYP) whereas experimental values are −5.13 and −2.84.^29^ In chlorobenzene (CB) solvent, the maximum absorption of IDFR for these functionals is computed *via* TD-DFT through the IEFPCM model, which is located at 369 (for ωB97XD), 456 (for MPW1PW91), 386 (for CAM-B3LYP), and 488 nm (for B3LYP) as presented in Fig. S1 (ESI†) whereas the experimental value is 440 nm. The aforementioned findings certified that the “MPW1PW91/6-31G (d, p)” DFT level proved to be in outstanding agreement with the experimental results. Therefore, the “MPW1PW91/6-31G (d, p)” theory is utilized for the investigation of the electronic, solubility, stability, optical, and charge-transporting properties of studied molecules. The electronic excitation analyses of investigated molecules are carried out at the selected theory and studied *via* Multiwfn 3.8 software.^34,35^ Utilizing Mulliken charge calculations, PyMOlyze 1.1 software^36^ executed a density-of-state analysis of the investigated molecules, and Origin 8.0 software^37^ was used for their graphical representation. According to the Marcus theory, the hole and electron hopping rate (*κ*_h_ and *κ*_e_) of studied molecules are calculated by using eqn (1) and (2).^38^1
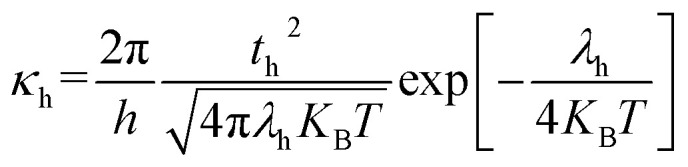
2
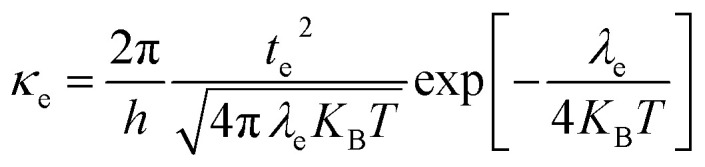
Here, *h* indicates Planck's constant, *t*_h_ shows hole transfer integral, *t*_e_ shows electron transfer integral, *λ*_h_ denotes hole reorganization energy, *λ*_e_ denotes electron reorganization energy, *K*_B_ indicates Boltzmann's constants, and *T* shows the room temperature. The *t*_h_ and *t*_e_ of investigated molecules are calculated *via*eqn (3) and (4)^39^ while *λ*_h_ and *λ*_e_ are computed by using eqn (5) and (6).^40^3
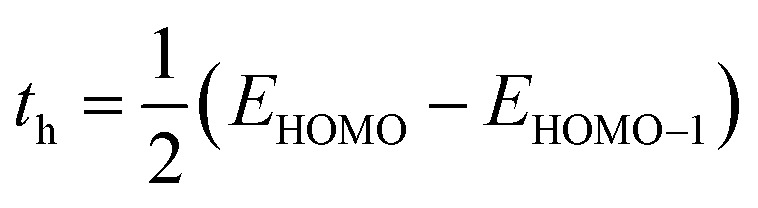
4
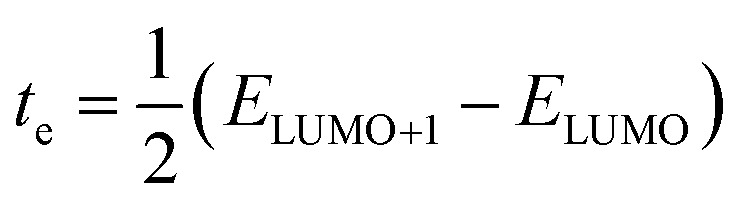

5 *λ*_h_ = [*E*^+^_0_ − *E*_+_] + [*E*^0^_+_ − *E*_0_]

6 *λ*_e_ = [*E*^−^_0_ − *E*_−_] + [*E*^0^_−_ – *E*_0_]

## 3. Results and discussions

### 3.1. Structural analysis

To evaluate the donor and hole-transport properties, all investigated molecules (IDFR, IDF1–IDF6, and Spiro-OMeTAD) are optimized at the “MPW1PW91/6-31G (d, p)” and their molecular geometries are shown in Fig. S2 (ESI†). The Cartesian coordinates of the ground state optimized structures of IDFR and IDF1–IDF6 molecules are presented in Tables S2–S8 (ESI†) while Cartesian coordinates of the Spiro-OMeTAD are presented in our previous work.^41^ In engineered molecules (IDF1–IDF6), the diethylene glycol chains are substituted at *N*-positions, and terthiophene-based side arms are substituted at the 2, 11-position of the fluorinated indolo[3,2-*b*]indole-based core, while end-capped acceptors are substituted on the *ortho* position of terthiophene. As given in Table 1, the dihedral angle (*θ*_1_) between core and diethylene glycol ranges from 83.54378° to 87.85649° while the dihedral angle (*θ*_2_) between core and terthiophene is 20.76515° to 21.95750° in IDFR and IDF1–IDF6 molecules. The dihedral angles (*θ*_3_ and *θ*_4_) across bithiophene–thiophene and thiophene-acceptor are 1.86032° to 5.83274° and 0.04399° to 0.49828° in the IDF1–IDF6 molecules. The bond length (*d*_1_) between core and diethylene glycol ranges from 1.44240 Å to 1.44326 Å while the bond length (*d*_2_) across core and terthiophene is 1.45707 Å to 1.45818 Å in IDFR and IDF1–IDF6 molecules. The bond length (*d*_3_) across bithiophene and thiophene is 1.43249 Å to 1.43585 Å and the bond length (*d*_4_) between thiophene-acceptor is 1.41141 Å to 1.42081 Å in the IDF1–IDF6 molecules whereas *d*_3_ between bithiophene and alkyl group is 1.49946 Å in the reference molecule. These findings are probably a result of the slightly tight π–π stacking in the designed molecules due to the addition of acceptors on the *ortho* position of bithiophene *via* thiophene, which allowed the improved donor (for OSCs) and hole-transporting properties (for PSCs) in our device.

**Table 1:** Dihedral angle and bond length between core and diethylene glycol (*θ*_1_ and *d*_1_), core and terthiophene (*θ*_2_ and *d*_2_), bithiophene and thiophene (*θ*_3_ and *d*_3_), thiophene and acceptor (*θ*_4_ and *d*_4_) of IDFR, and IDF1–IDF6 molecules

Molecules	*θ*_1_ (°)	*θ*_2_ (°)	*θ*_3_ (°)	*θ*_4_ (°)	*d*_1_ (Å)	*d*_2_ (Å)	*d*_3_ (Å)	*d*_4_ (Å)
IDFR	83.54378	21.95750	—	—	1.44240	1.45818	1.49946	—
IDF1	87.85649	21.63553	5.83274	0.37233	1.44330	1.45737	1.43518	1.41830
IDF2	85.03097	21.36875	5.07842	0.04399	1.44303	1.45733	1.43502	1.41639
IDF3	85.05367	21.05826	2.54271	0.22604	1.44297	1.45733	1.43473	1.41582
IDF4	84.80784	21.10916	1.93264	0.07018	1.44326	1.45707	1.43249	1.41141
IDF5	84.49925	21.33767	3.15670	0.12655	1.44294	1.45736	1.43570	1.41861
IDF6	85.17401	20.76515	1.86032	0.49828	1.44307	1.45742	1.43585	1.42081

**Fig. 2:**
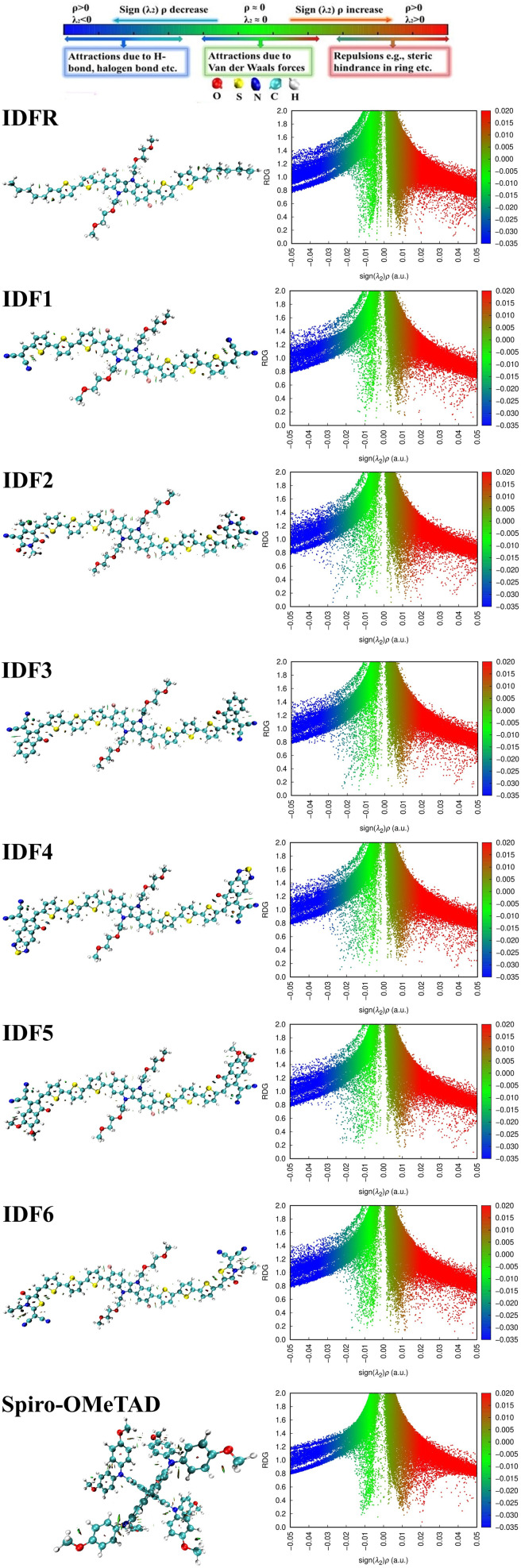
NCI isosurfaces and 2D-RDG plots of investigated molecules.

Furthermore, the interactions present in the studied molecules (IDFR, IDF1–IDF6, and Spiro-OMeTAD) are assessed *via* non-covalent interactions (NCI), and their isosurfaces which are plotted by using Multiwfn 3.8 software. NCI plots demonstrate repulsive and attractive interactions among various regions of the molecule that depend on a reduced density gradient (RDG) and electron density (*r*) whereas isosurfaces show various interactions *via* electron density. As shown in Fig. 2, NCL plots show the existence of attractive non-covalent interactions in the investigated molecules, showing green-colored peaks spreading to the left and negative values of the effective density “sign(*λ*_2_)*ρ*” revealing electron depletion regions. The van der Waals forces are more dominant in engineered molecules than reference (IDFR) and Spiro-OMeTAD, which are responsible for improving molecular stability. Moreover, the investigated molecules possessed substantial red and green isosurface patches that reveal the repulsive and attractive forces, in which repulsions push them out of the plane and attractive interactions try to keep it in the plane.

### 3.2. Electronic property

**Fig. 3:**
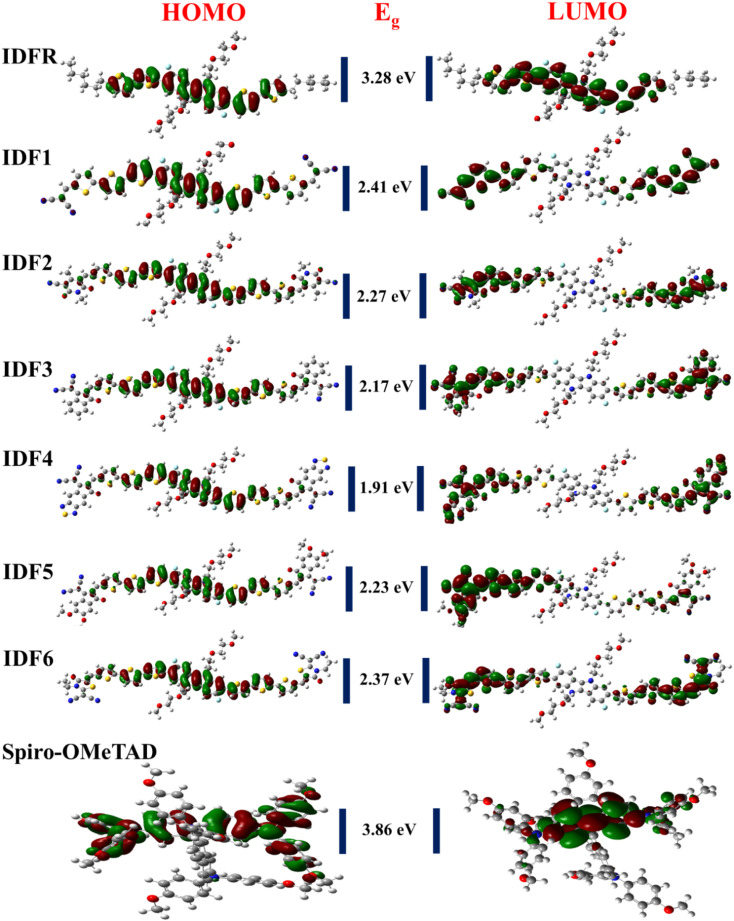
HOMO and LUMO densities with bandgaps (*E*_g_) of studied molecules.

In PSCs, the matched energy levels across the perovskite material, electron-transporting material, and hole-transporting material are essential for facilitating the electron and hole extraction at the interface,^25,26^ and deeper HOMO levels with low bandgaps are essential in OSCs,^42^ which enhance their photovoltaic properties, open circuit voltage, and efficiency. The HOMO and LUMO levels (*E*_HOMO_ and *E*_LUMO_) of studied molecules are calculated from their ground-state optimized geometries. As presented in Table 2, *E*_HOMO_ of the studied molecules is −4.96 eV (IDFR), −5.46 eV (IDF1), −5.42 eV (IDF2), −5.38 eV (IDF3), −5.50 eV (IDF4), −5.31 eV (IDF5), −5.37 eV (IDF6), and −4.47 eV (Spiro-OMeTAD) while *E*_LUMO_ is −1.68 eV (IDFR), −3.05 eV (IDF1), −3.15 eV (IDF2), −3.21 eV (IDF3), −3.59 eV (IDF4), −3.08 eV (IDF5), −3.00 eV (IDF6), and −0.61 eV (Spiro-OMeTAD), respectively. The findings indicated that the addition of acceptors on the *ortho* position of bithiophene *via* thiophene in the reference (IDFR) molecule enhances HOMO levels of engineered molecules (IDF1–IDF6) than the reference (IDFR) and Spiro-OMeTAD, which are beneficial for effective hole injection from the perovskite layer, relative to the valence band maximum of the MAFAPbI_3_ (−5.91 eV)^43^ in PSCs. The band gap (*E*_LUMO_ − *E*_HOMO_) of investigated molecules is 3.28 (IDFR), 2.41 (IDF1), 2.27 (IDF2), 2.17 (IDF3), 1.91 (IDF4), 2.23 (IDF5), 2.37 (IDF6), and 3.86 eV (Spiro-OMeTAD). FMOs (HOMO and LUMO) with bandgaps (*E*_g_) of studied molecules are indicated in Fig. 3. The deeper HOMO levels with low bandgaps of designed molecules enhance the open-circuit photovoltage and efficiency of OSCs and PSCs.

**Table 2:** Band gap (*E*_g_), HOMO, and LUMO energies with orbital contribution between fragments of studied molecules (IDFR, IDF1–IDF6, and Spiro-OMeTAD)

Molecules	*E*_g_ (eV)	Orbitals	Energy (eV)	Core (eV)	Thiophene (eV)	DEG/DPA unit (eV)	Acceptor (eV)
IDFR	3.28	HOMO	−4.96	50.3	47.8	0.9	—
		LUMO	−1.68	49.0	49.6	0.6	—
IDF1	2.41	HOMO	−5.46	50.5	44.7	1.2	3.7
		LUMO	−3.05	4.4	58.7	0.0	36.8
IDF2	2.27	HOMO	−5.42	45.6	47.3	1.0	6.2
		LUMO	−3.15	3.1	47.5	0.0	49.4
IDF3	2.17	HOMO	−5.38	46.0	47.2	1.0	5.8
		LUMO	−3.21	2.1	38.5	0.0	59.3
IDF4	1.91	HOMO	−5.50	47.8	44.7	1.1	6.5
		LUMO	−3.59	1.3	30.0	0.0	68.7
IDF5	2.23	HOMO	−5.31	43.5	49.4	0.9	6.2
		LUMO	−3.08	2.0	36.7	0.0	61.3
IDF6	2.37	HOMO	−5.37	46.7	47.1	0.9	5.3
		LUMO	−3.00	3.4	48.2	0.0	48.4
Spiro-OMeTAD	3.86	HOMO	−4.47	33.4	—	60.7	—
		LUMO	−0.61	83.8	—	15.8	—

Moreover, the density of state (DOS) and overlap population DOS (OPDOS) analyses are effective tools for studying the electronic configuration of molecules and their impact on electrical and optical properties.^44,45^ The DOS analysis shows the molecule fragment's contribution to HOMO and LUMO energies formation.^46^ In the DOS graph, the positive values indicate LUMO energies and negative values show HOMO energies on the *x*-axis. The OPDOS analysis reveals the relation between chemical bonding and overlap population, allowing for the evaluation of bonding, anti-bonding, and non-bonding interactions between a molecule's orbitals.^47^ The zero value indicates non-bonding, the positive value indicates bonding and the negative value shows anti-bonding interactions between various orbitals. The DOS and OPDOS analyses of studied molecules are performed at the “MPW1PW91/6-31G (d, p)” level and manipulated by using the PyMolyze 1.1 program. The designed molecules (IDF1–IDF6) consist of the fluorinated indolo[3,2-*b*]indole (core), diethylene glycol (DEG unit), terthiophene (thiophene), and end-capped acceptors whereas the reference molecule (IDFR) consists of the fluorinated indolo[3,2-*b*]indole (core), diethylene glycol (DEG unit), bithiophene (thiophene), alkyl unit and Spiro-OMeTAD molecule composed of spirobifluorene core, diphenylamine (DPA unit), and methoxy group.

**Fig. 4:**
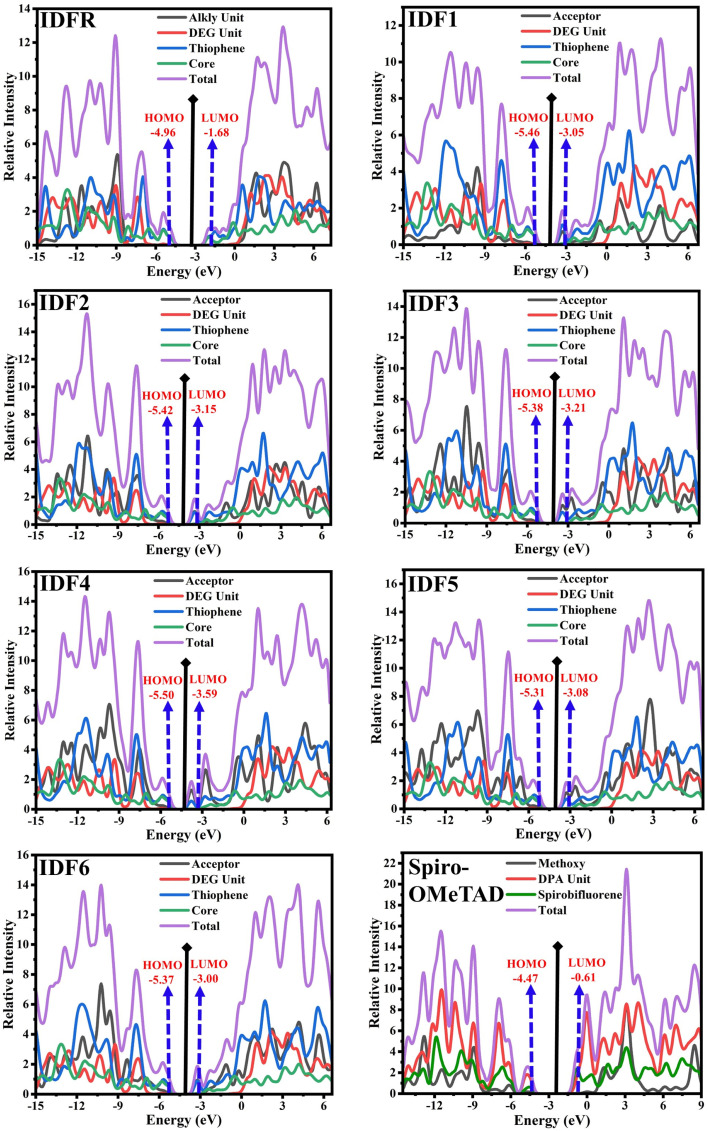
DOS graphs of studied molecules (IDFR, IDF1–IDF6, and Spiro-OMeTAD) reveal the molecule fragment's contribution to the formation of HOMO and LUMO levels.

**Fig. 5:**
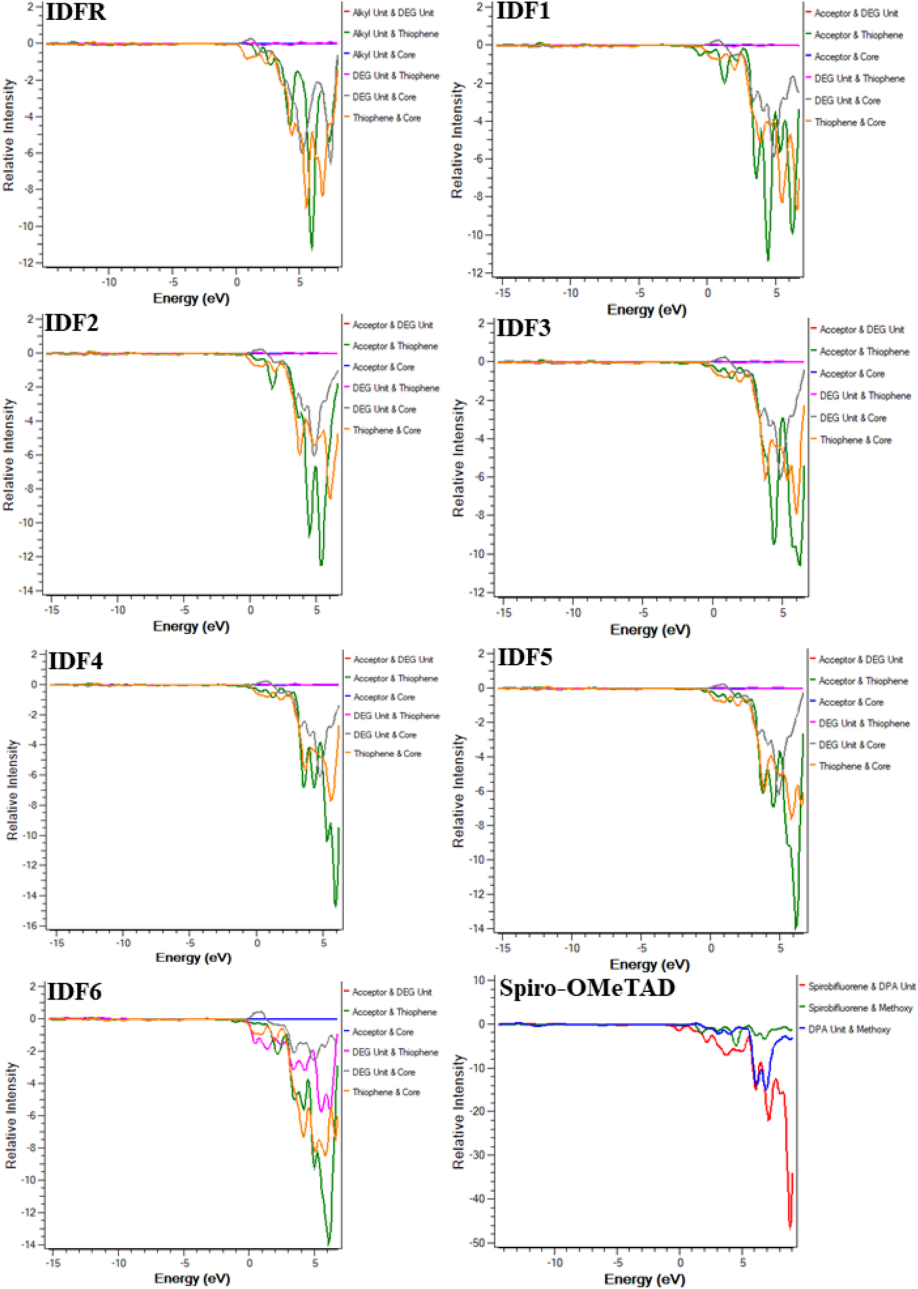
OPDOS plots of studied molecules show interactions among fragments.

The DOS calculations and graphs of studied molecules (IDFR, IDF1–IDF6, and Spiro-OMeTAD) are indicated in Table 2 and Fig. 4. As presented in Table 2, the HOMO levels of engineered molecules (IDF1–IDF6) are primarily formed *via* core (43.5 to 50.5 eV) and terthiophene (44.7 to 49.4 eV) and the LUMO levels are majorly formed *via* terthiophene (30.0 to 58.7 eV) and end-capped acceptor (36.8 to 68.7 eV). The HOMO and LUMO energies of IDFR are majorly formed *via* core (50.3 eV for HOMO, 49.0 eV for LUMO) and bithiophene (47.8 eV for HOMO, 49.6 eV for LUMO) while spirobifluorene core (33.4 eV for HOMO, 83.8 eV for LUMO) and diphenylamine DPA unit (60.7 eV for HOMO, 15.8 eV for LUMO) fragments are majorly contributed in the formation of HOMO and LUMO energies of Spiro-OMeTAD molecule. The aforementioned findings certified that the charge transfer from fluorinated indolo[3,2-*b*]indole core to acceptors *via* the extended conjugation system in engineered molecules and fluorinated indolo[3,2-*b*]indole core to bithiophene in reference molecule while diphenylamine to spirobifluorene in the Spiro-OMeTAD molecule. As indicated in Fig. 5, the designed molecules indicate the highest interaction between thiophene & fluorinated indolo[3,2-*b*]indole-based core and acceptor & thiophene. The IDFR demonstrates the highest interaction across thiophene & fluorinated indolo[3,2-*b*]indole-based core and alkyl unit & thiophene whereas Spiro-OMeTAD shows maximum interaction between spirobifluorene core and diphenylamine DPA unit. The results showed that the engineered molecules interact more in donor–acceptor portions, indicating that they effectively contribute to the performance of solar cells.

### 3.3. Optical property

**Fig. 6:**
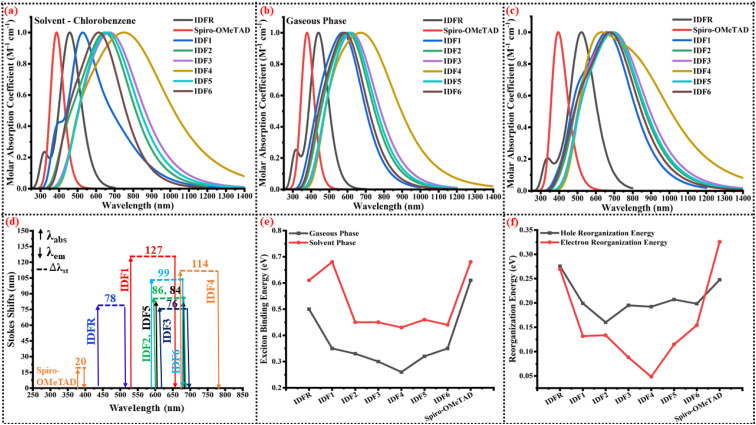
(a) Normalized UV-vis spectra in the solvent phase. (b) Normalized UV-vis spectra in the gaseous phase. (c) Normalized emission spectra. (d) Stokes shifts. (e) Exciton binding energy. (f) Reorganization energy of investigated molecules (IDFR, IDF1–IDF6, and Spiro-OMeTAD).

To access the influence of acceptor in engineered molecules (IDF1–IDF6), the optical properties of investigated molecules are computed at “MPW1PW91/6-31G (d, p)” TD-DFT level based on the ground-optimized and excited-state geometries, and findings are presented in Table 3. The maximum absorption (*λ*_max_) of studied molecules is located at 442 nm (IDFR), 529 nm (IDF1), 600 nm (IDF2), 620 nm (IDF3), 670 nm (IDF4), 609 nm (IDF5), 581 nm (IDF6), 377 nm (Spiro-OMeTAD) in gaseous phase and 456 nm (IDFR), 530 nm (IDF1), 645 nm (IDF2), 674 nm (IDF3), 748 nm (IDF4), 660 nm (IDF5), 620 nm (IDF6), 387 nm (Spiro-OMeTAD) in chlorobenzene (CB) solvent phase, as indicated in Fig. 6a and b, respectively. The designed molecules (IDF2–IDF6) show distinct bathochromic shifts from the reference (IDFR) and Spiro-OMeTAD, demonstrating that adding acceptor units may effectively decrease the band gap and improve intramolecular electrical interactions. In PSCs, the engineered molecules should be transparent in the visible light region and have no absorption screening effect towards the perovskite layer, as observed in previous studies^48–50^ while bathochromic shifts of IDF2–IDF6 molecules cause absorption of broad-range radiations which enhance the efficiency of OSCs.

**Table 3:** Properties of investigated molecules (IDFR, IDF1–IDF6, and Spiro-OMeTAD)

Molecules	λ^gas^_abs_ a	*λ*_em_ b	Δ*λ*_st_^c^	λ^solv^_abs_ d	*f* e	LHE^f^	Assignment^g^	*E* ^gas^_b_ h	*E* ^solv^_b_ i	EA^j^	IP^k^	*η* l	Δ*G*_solv_^m^	*V*_OC_ n
IDFR	442	520	78	456	2.13	0.993	H → L (70%)	0.50	0.61	0.84	5.81	2.49	−9.97	0.96
IDF1	529	656	127	530	2.34	0.995	H → L (69%)	0.35	0.68	2.38	6.18	1.90	−20.15	1.46
IDF2	600	686	86	645	2.74	0.998	H → L (69%)	0.33	0.45	2.53	6.09	1.78	−23.91	1.42
IDF3	620	696	76	674	2.59	0.997	H → L (69%)	0.30	0.45	2.61	6.05	1.72	−19.84	1.38
IDF4	670	784	114	748	2.07	0.992	H → L (69%)	0.26	0.43	3.03	6.15	1.56	−21.51	1.50
IDF5	609	693	84	660	2.78	0.998	H → L (63%)	0.32	0.46	2.50	5.96	1.73	−22.12	1.31
IDF6	581	680	99	620	2.65	0.998	H → L (67%)	0.35	0.44	2.39	6.05	1.83	−21.53	1.37
Spiro-OMeTAD	377	397	20	387	0.08	0.168	H → L (60%)	0.61	0.68	1.09	5.11	2.01	−15.54	0.47

a Maximum absorption in the gaseous phase (in nm).

b Maximum emission in the gaseous phase (in nm).

c Stokes shifts (in nm).

d Maximum absorption in the solvent phase (in nm).

e Oscillator strength.

f Light-harvesting efficiency.

g Main orbital contributions.

h Binding energy in the gaseous phase (in eV).

i Binding energy in the solvent phase (in eV).

j Electron affinity (in eV).

k Ionization potential (in eV).

l Absolute hardness (in eV).

m Solvation-free energy (in kcal mol^−1^).

n Open-circuit voltage (in V).

Recently studies suggested that the large Stokes shifts and lower *T*_g_ molecules facilitated the infiltration and pore-filling ability of HTMs for PSCs.^51^ The substantial Stokes shift (Δ*λ*_st_) indicates a significant structural change from the ground to its first excited states, suggesting that the HTMs may be flexible and beneficial for the light soaking post-treatment. The Δ*λ*_st_ of investigated molecules are calculated *via* the differences in maximum emission (*λ*_em_) and absorption (*λ*_abs_). As given in Table 3, the maximum emission (*λ*_em_) of studied molecules is 520 nm (IDFR), 656 nm (IDF1), 686 nm (IDF2), 696 nm (IDF3), 784 nm (IDF4), 693 nm (IDF5), 680 nm (IDF6), and 397 nm (Spiro-OMeTAD) as indicated in Fig. 6(c). The Δ*λ*_st_ of investigated molecules is 78 nm (IDFR), 127 nm (IDF1), 86 nm (IDF2), 76 nm (IDF3), 114 nm (IDF4), 84 nm (IDF5), 99 nm (IDF6), and 20 nm (Spiro-OMeTAD) as presented in Fig. 6d. The IDF1 and IDF4 molecules show large Stokes shifts due to the π-extended cyano- and thiodiamine indan-based end-capped acceptors. Overall results indicated that engineered molecules (except IDF3) are more flexible and beneficial for the light soaking post-treatment compared to IDFR molecule.

During the process of optical excitation, the exciton binding energy (*E*_b_) is an essential tool to assess the charge transfer properties of the studied molecules which is computed *via* the difference of band gap (*E*_g_) and first excitation energy (*E*_x_).^52^ The *E*_x_ (in eV) of investigated molecules is 2.78 (IDFR), 2.06 (IDF1), 1.94 (IDF2), 1.87 (IDF3), 1.65 (IDF4), 1.91 (IDF5), 2.02 (IDF6), 3.25 (Spiro-OMeTAD) in gaseous phase and 2.67 (IDFR), 1.73 (IDF1), 1.82 (IDF2), 1.72 (IDF3), 1.48 (IDF4), 1.77 (IDF5), 1.93 (IDF6), 3.18 (Spiro-OMeTAD) in solvent (chlorobenzene) phase. As shown in Table 3 and Fig. 6e, the *E*_b_ (in eV) of studied molecules is 0.50 (IDFR), 0.35 (IDF1), 0.33 (IDF2), 0.30 (IDF3), 0.26 (IDF4), 0.32 (IDF5), 0.35 (IDF6), 0.61 (Spiro-OMeTAD) in gaseous phase and 0.61 (IDFR), 0.68 (IDF1), 0.45 (IDF2), 0.45 (IDF3), 0.43 (IDF4), 0.46 (IDF5), 0.44 (IDF6), 0.68 (Spiro-OMeTAD) in solvent (chlorobenzene) phase, respectively. The designed molecules (except IDF1 in the solvent phase) indicated a smaller *E*_b_ compared to IDFR and Spiro-OMeTAD, indicating that the electron and hole easily dissociate into positive and negative charges and enhance their charge transfer properties. Moreover, the results of oscillator strength and light-harvesting efficiency indicated that the engineered molecules (except IDF4) have a higher photocurrent flow ability than the IDFR molecule, resulting in their high potential for the manufacturing of high-efficiency OSCs and PSCs.

### 3.4. Electronic excitation analyses

**Fig. 7:**
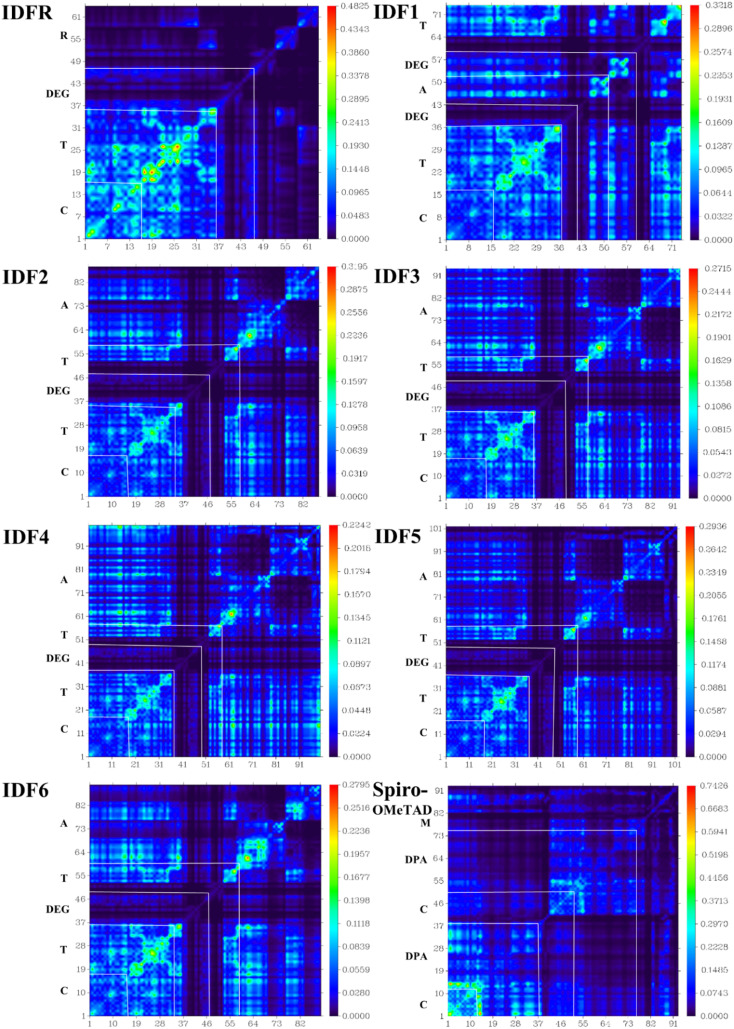
Transition density matrix (TDM) maps of studied molecules.

In charge transporting materials, efficient charge transfer through electronic transitions leads to boosting the charge mobility with low recombination losses, which are effective for substantial hole extraction and high transportation to the electrode. Therefore, electronic excitation analyses *i.e.*, transition density matrix (TDM), inter-fragment charge transfer (IFCT), and hole–electron distribution/overlap (HED) analyses are crucial for understanding and visualizing charge excitation, diffusion, separation processes, and recombination in the organic molecules.^44,53,54^ The electronic excitation analyses of investigated molecules are carried out at the selected theory and studied *via* Multiwfn 3.8 software. To illustrate the TDM and HED analyses, we divided IDF1–IDF6 molecules into the fluorinated indolo [3,2-*b*] indole core (C), diethylene glycol (DEG), terthiophene (T), and end-capped acceptors (A) fragments whereas the reference (IDFR) consisted of the fluorinated indolo [3,2-*b*] indole core (C), diethylene glycol (DEG), bithiophene (T), alkyl unit (*R*) and Spiro-OMeTAD molecule composed of spirobifluorene core (C), diphenylamine (DPA), and methoxy group (M). As shown in Fig. 7, IDF1–IDF6 molecules indicated effective charge coherence and maximum charge density from core to terthiophene and end-capped acceptors whereas IDFR (reference) molecule demonstrated charge density from core to terthiophene and Spiro-OMeTAD molecule showed in the portion of spirobifluorene core to diphenylamine. As a result, engineered molecules show stronger exciton dissociation with sharper charge flow as compared to IDFR and Spiro-OMeTAD molecules.

**Fig. 8:**
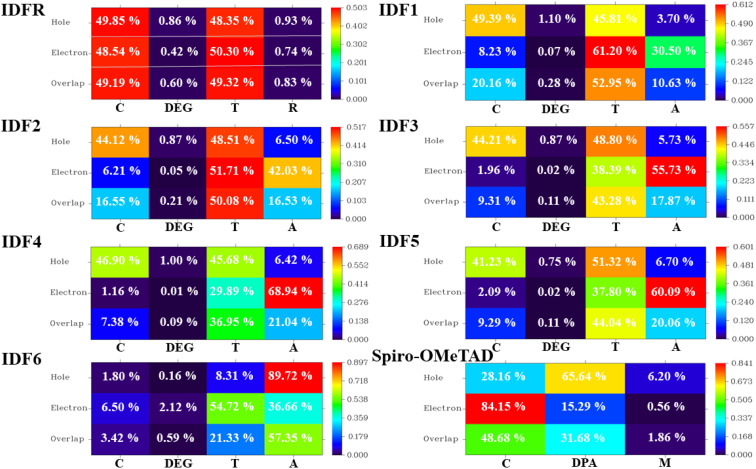
Hole–electron distribution (HED) heat maps with percentage contribution to spatial charge production and overlap of investigated molecules (IDFR, IDF1–IDF6, and Spiro-OMeTAD).

The HED analysis indicated that the IDFR molecule (reference) has a substantial amount of overlapping (99.91%) across holes and electrons, which increases local charge excitations and decreases the charge transfer rate and photocurrent density. Due to end-capped acceptor moiety engineering, the amount of overlapping across holes and electrons decreased significantly (99.91% to 65.46–84.02%), resulting in a high charge transfer rate and photocurrent density with low local charge excitations in designed molecules (IDF1–IDF6) as indicated in Fig. 8. The designed molecules show high hole generation in the fluorinated indolo [3,2-*b*] indole & thiophene portion and electron generation in the thiophene & acceptor portion while the reference molecule has hole and electron generation in the fluorinated indolo [3,2-*b*] indole & thiophene portion. Spiro-OMeTAD molecule has 82.22% electron–hole overlapping and high hole and electron generation in the spirobifluorene and diphenylamine portion. As shown in Table S1†, the IFCT analysis demonstrated that the engineered molecules show maximum intrinsic charge transfer (62.437% to 81.374%) and minimum intrinsic local excitation (18.626% to 37.563%) than the reference molecule (ICT = 51.471% and ILE = 48.529%), resulting low charge losses with high charge-density flow. Overall, electronic excitation analyses of studied molecules showed that the engineered molecules show stronger exciton dissociation, low charge coupling, and high intrinsic charge transfer with sharper charge flow as compared to IDFR and Spiro-OMeTAD molecules.

### 3.5. Solubility and stability

To obtain a better morphology of molecules in OSCs and PSCs, the solubility of the investigated molecules is a crucial parameter, which is usually accessed by the solvation Gibbs free energy (Δ*G*_solv_ = *G*_sol_ − *G*_gas_).^55^ The efficiency of the material solution increases with the deeper values of solvation-free energy (Δ*G*_solv_). As presented in Table 3, Δ*G*_solv_ (in kcal mol^−1^) of studied molecules is −9.97 (IDFR), −20.15 (IDF1), −23.91 (IDF2), −19.84 (IDF3), −21.51 (IDF4), −22.12 (IDF5), 21.53 (IDF6), and −15.54 (Spiro-OMeTAD). Among them, the IDF2 molecule exhibits a deeper value of solvation Gibbs free energy (−23.91 kcal mol^−1^), showing that the addition of pyridine-based strong electron-donating side arms is beneficial in the facilitation of small molecule solutions. Overall results indicate that designed molecules are probably more soluble than Spiro-OMeTAD and IDFR (reference) molecules, indicating that it will facilitate in the manufacturing of OSCs and PSCs.

**Fig. 9:**
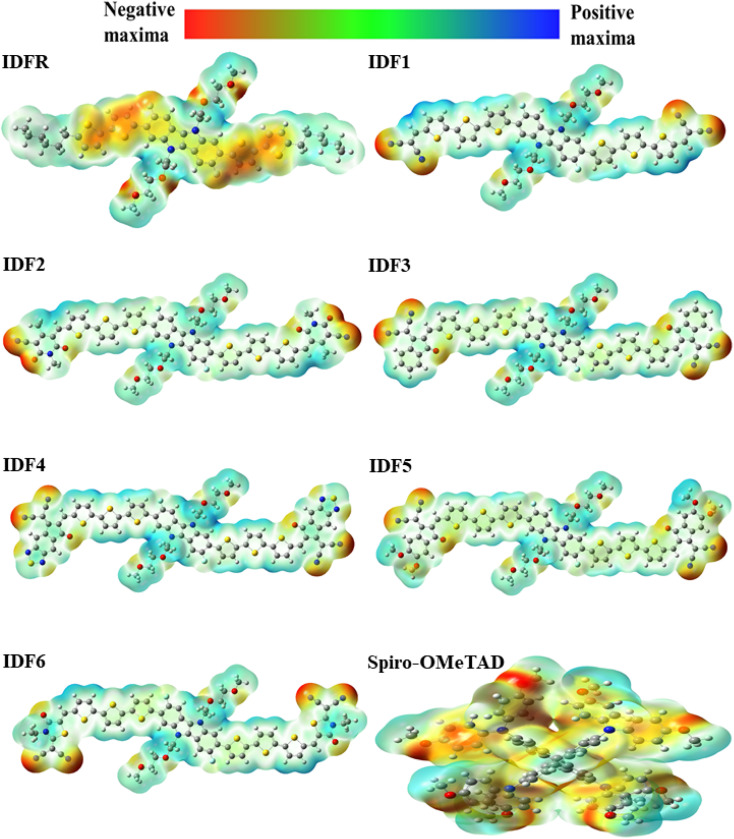
Molecular electrostatic potential (ESP) surfaces of IDFR, IDF1–IDF6, and Spiro-OMeTAD molecules.

The absolute hardness 
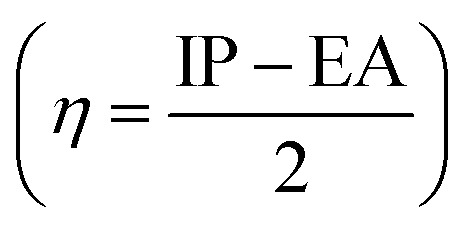
 and the molecular electrostatic potential (ESP) are computed to evaluate the stability of the investigated system. The greater value of absolute hardness (*η*) indicates higher material stability. As indicated in Table 3, the *η* value (in eV) of the investigated molecules is 2.49 (IDFR), 1.90 (IDF1), 1.78 (IDF2), 1.72 (IDF3), 1.56 (IDF4), 1.73 (IDF5), 1.83 (IDF6), and 2.01 (Spiro-OMeTAD), respectively. The values of electron affinity, which is greater in designed molecules (2.38 to 3.03 eV) compared to the reference (0.84 eV) and Spiro-OMeTAD (1.09 eV) molecules, are primarily responsible for the difference in absolute hardness (*η*). The calculated findings of *η* demonstrated that the engineered molecules are less stable than the IDFR and comparable to the Spiro-OMeTAD. Furthermore, the ESP analysis provides useful information about molecule stability with suitable regions for molecular attacks. The ESP maps of studied molecules are presented in Fig. 9 in which the green color demonstrates the neutral portion, the red denotes the negative charge, and the blue color denotes the positive charge. In designed molecules (IDF1–IDF6), the positive charges are dominated on the fluorinated indolo[3,2-*b*]indole core, diethylene glycol (except oxygen atoms), and terthiophene, which are vulnerable to the nucleophile, while the negative charges are located on oxygen and nitrogen atoms, which are vulnerable to the electrophile. The IDFR molecule has majorly negative charges on fluorinated indolo[3,2-*b*]indole core & oxygen atoms and positive charges on the alkyl unit & diethylene glycol (except oxygen atoms) whereas the Spiro-OMeTAD molecule has majorly negative charges on the spirobifluorene and diphenylamine portion.

### 3.6. Charge transport property

Designing molecules with high carrier mobility is crucial for the manufacturing of highly effective OSCs and PSCs. Therefore, obtaining high electron and hole mobility is the most crucial step in the designing of molecules. Herein, the addition of the acceptor approach in the A–D–A typed scaffold is assessed. According to the Marcus theory, the hole and electron hopping rate (*κ*_h_ and *κ*_e_) of molecules are influenced *via* two crucial parameters of hole and electron reorganization energy (*λ*_h_ and *λ*_e_) and transfer integral (*t*_h_ and *t*_e_).^56^ The small *λ*_h_ and *λ*_e_ with large *t*_h_ and *t*_e_ is in favor of improving the charge transport in molecules. The reorganization energy is usually calculated by the contribution of internal nuclear reorganization and external polarized energy. Based on polarized force field calculations, the external polarized energy is less than that of the nuclear part, and its influence on the charge transport is negligible. As a result, the internal *i.e.*, hole and electron reorganization energies (*λ*_h_ and *λ*_e_) is computed by using eqn (5) and (6). As indicated in Table 4 and Fig. 6f, the *λ*_h_ value (in eV) of studied molecules is 0.2757 (IDFR), 0.1992 (IDF1), 0.1602 (IDF2), 0.1950 (IDF3), 0.1924 (IDF4), 0.2071 (IDF5), 0.1984 (IDF6), 0.2476 (Spiro-OMeTAD) and *λ*_e_ value (in eV) is 0.2694 (IDFR), 0.1318 (IDF1), 0.1336 (IDF2), 0.0881 (IDF3), 0.0484 (IDF4), 0.1148 (IDF5), 0.1542 (IDF6), 0.3256 (Spiro-OMeTAD). The designed molecules have lower *λ*_h_ than the IDFR and Spiro-OMeTAD, especially the IDF2, IDF3, and IDF4 molecules which show the addition of acceptor is a better design approach to facilitating hole transfer and are expected to be the future molecules for the manufacturing of high-efficiency OSCs and PSCs.

**Table 4:** Hole and electron reorganization energy (*λ*_h_ and *λ*_e_), hole and electron transfer integral (*t*_h_ and *t*_e_), hole and electron hopping rate (*κ*_h_ and *κ*_e_), and total amount of charge transfer (Δ*N*_max_) of IDFR, IDF1–IDF6, and Spiro-OMeTAD molecules

Molecules	*λ*_h_ (eV)	*λ*_e_ (eV)	*t*_h_ (eV)	*t*_e_ (eV)	*κ*_h_ (s^−1^)	*κ*_e_ (s^−1^)	Δ*N*_max_ (e)
IDFR	0.2757	0.2694	0.2581	0.2618	1.1456 × 10^13^	3.3589 × 10^13^	1.33
IDF1	0.1992	0.1318	0.2205	0.0237	2.2686 × 10^14^	5.9376 × 10^12^	2.24
IDF2	0.1602	0.1336	0.1861	0.0173	2.6808 × 10^14^	3.1040 × 10^12^	2.41
IDF3	0.1950	0.0881	0.1909	0.0128	1.8089 × 10^14^	2.7466 × 10^12^	2.50
IDF4	0.1924	0.0484	0.1992	0.0090	2.0441 × 10^14^	2.2126 × 10^12^	2.91
IDF5	0.2071	0.1148	0.1785	0.0268	1.3140 × 10^14^	9.0738 × 10^12^	2.42
IDF6	0.1984	0.1542	0.1933	0.0181	1.7614 × 10^14^	2.7151 × 10^12^	2.29
Spiro-OMeTAD	0.2476	0.3256	0.1420	0.0310	3.3313 × 10^13^	2.0365 × 10^12^	1.26

Eqn (3) and (4) are utilized to calculate the hole and electron transfer integral (*t*_h_ and *t*_e_) which shows internal molecule stacking. As presented in Table 4, the *t*_h_ value (in eV) of studied molecules is 0.2581 (IDFR), 0.2205 (IDF1), 0.1861 (IDF2), 0.1909 (IDF3), 0.1992 (IDF4), 0.1785 (IDF5), 0.1933 (IDF6), 0.1420 (Spiro-OMeTAD) and *t*_e_ value (in eV) is 0.2618 (IDFR), 0.0237 (IDF1), 0.0173 (IDF2), 0.0128 (IDF3), 0.0090 (IDF4), 0.0268 (IDF5), 0.0181 (IDF6), 0.0310 (Spiro-OMeTAD). The findings demonstrated that engineered molecules have a lower hole transfer integral than the IDFR (reference) but higher than the Spiro-OMeTAD. The hole and electron hopping rate (*κ*_h_ and *κ*_e_) of studied molecules that are mainly influenced by the hole and electron reorganization energy (*λ*_h_ and *λ*_e_) and transfer integral (*t*_h_ and *t*_e_) are computed by using eqn (1) and (2). As presented in Table 4, the hole hopping rate of studied molecules is in the range of 1.1456 × 10^12^ to 2.6808 × 10^14^ s^−1^, and the electron hopping rate is in the range of 2.0365 × 10^12^ to 3.3589 × 10^13^ s^−1^. The results demonstrated that engineered molecules have a higher hole hopping rate than the IDFR and Spiro-OMeTAD due to the substitution of acceptor on the *ortho* position of bithiophene *via* thiophene in IDFR. In addition, the total amount of charge transfer (Δ*N*_max_) also reveals the charge transport ability of investigated molecules.^57^ As presented in Table 4, the Δ*N*_max_ of studied molecules is 1.33 (IDFR), 2.24 (IDF1), 2.41 (IDF2), 2.50 (IDF3), 2.91 (IDF4), 2.42 (IDF5), 2.29 (IDF6), and 1.26*e* (Spiro-OMeTAD). The findings demonstrated that engineered molecules have a higher Δ*N*_max_ value than reference IDFR and Spiro-OMeTAD molecules, which improves their charge transfer ability. In conclusion, the high hole hopping rate, high total amount of charge transfer, and low reorganization energy with comparable charge transfer integral demonstrated that engineered molecules have an efficient hole transport potential for photovoltaic cells.

### 3.7. Open circuit voltage

The open-circuit voltage (*V*_OC_) is a crucial parameter for measuring the efficiency of OSCs and PSCs that is produced by a solar cell when no current is passing through it.^39^ The matched and more negative HOMO levels of molecules across the perovskite material are beneficial for effective hole injection from the perovskite layer while more negative HOMO levels with low bandgaps are essential for OSCs, which enhance their photovoltaic properties, open circuit voltage, and efficiency.^42,58^ Other factors such as the interface energetics, film morphology, and charge recombination kinetics also play important roles in V_OC_ evaluation.^59,60^ Theoretically, Scharber's eqn (7) is used to estimate the *V*_OC_ of IDFR, IDF1–IDF6, and Spiro-OMeTAD molecules in which *e* indicates the charge on the molecule and 0.3 demonstrates the offset in charge generation at the molecular interface.^61^7
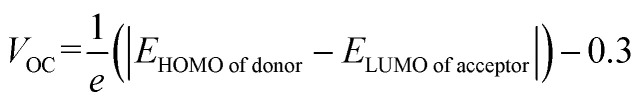


To satisfy Scharber's equation, the HOMO energy levels of the investigated molecules are compared with the LUMO level of the popular acceptor PC_61_BM (HOMO = −6.10 eV, LUMO = −3.70 eV).^39^ As indicated in Table 3, the *V*_OC_ values of studied molecules are 0.96 V (IDFR), 1.46 V (IDF1), 1.42 V (IDF2), 1.38 V (IDF3), 1.50 V (IDF4), 1.31 V (IDF5), 1.37 V (IDF6), and 0.47 V (Spiro-OMeTAD). The increasing order of estimated V_OC_ for the studied molecules is Spiro-OMeTAD < IDFR < IDF5 < IDF6 < IDF3 < IDF2 < IDF1 < IDF4. The results indicated that the addition of an acceptor increased the *V*_OC_ of engineered molecules than IDFR and Spiro-OMeTAD molecules which enhanced the efficiency of PSCs and OSCs. The calculated HOMO levels of engineered molecules are close to the threshold levels of the perovskite layer but do not exceed them, so IDF1–IDF6 provide an effective driving force for hole extraction out of the perovskite layer and high *V*_OC_. For OSCs, an exceptionally higher *V*_OC_ of 1.31 to 1.50 V has been explored for the highest combination IDF1–IDF6 : PC_61_BM. The low laying energy levels of the engineered molecules show a perfect matching of hole and electron transporting interfaces. Hence, the designed molecules (IDF1–IDF6) manifest the exciting potential to be employed as novel materials in high-performance photovoltaic devices.

### 3.8. Synthetic pathway

**Fig. 10:**
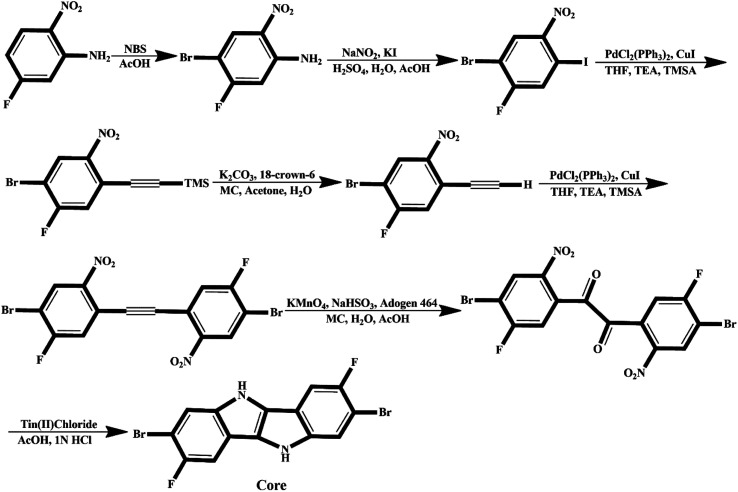
Suggested synthetic pathway for fluorinated indolo[3,2-*b*]indole core.

**Fig. 11:**
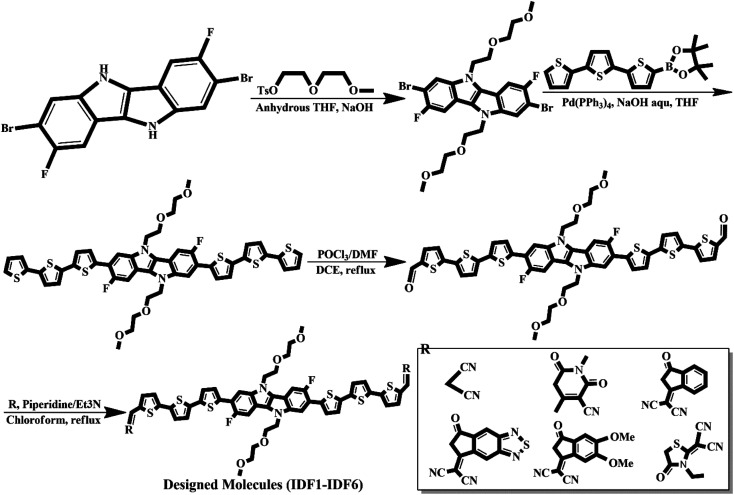
Suggested synthetic pathway for engineered IDF1–IDF6 molecules.

We believe that engineered molecules (IDF1–IDF6) can be synthesized experimentally, and the suggested synthetic pathway is shown in Fig. 11. The synthetic process of fluorinated indolo[3,2-*b*]indole core is shown in Fig. 10 and described in the previous study.^62^ The core is substituted with diethylene glycol chains at *N*-positions and terthiophene conjugated arms at 2-,7-position *via* S_N_2 reaction and Suzuki cross-coupling reaction,^29,63^ then the acceptors are introduced with piperidine/Et3N, chloroform, and reflux^63^ to synthesize the engineered molecules (IDF1–IDF6).

## 4. Conclusion

In summary, six molecules namely IDF1, IDF2, IDF3, IDF4, IDF5, and IDF6 are designed which are based on the fluorinated indolo[3,2-*b*]indole-based core with diethylene glycol chains, terthiophene-based side arms and end-capped acceptors with A–D–A configuration. The results demonstrated that IDF1–IDF6 molecules have tight π–π stacking, more negative HOMO levels (−5.50 to −5.31 eV), low bandgaps (1.91 to 2.41 eV), high absorption coefficient, large Stokes shifts, high open-circuit photovoltages (1.31 to 1.50 V), and superior solubility with comparable stability than the reference (IDFR) and Spiro-OMeTAD. The results of oscillator strength (*f*) and light-harvesting efficiency (LHE) indicated that the engineered molecules (except IDF4) have a higher photocurrent flow ability than the IDFR molecule, resulting in their high potential for the manufacturing of high-efficiency OSCs and PSCs. The TDM analysis indicated that the engineered molecules show stronger exciton dissociation with sharper charge flow than the IDFR and Spiro-OMeTAD molecules. Due to end-capped acceptor moiety engineering, the amount of overlapping across holes and electrons decreased significantly (99.91% to 65.46–84.02%), resulting in a high charge transfer rate and photocurrent density with low local charge excitations in the designed molecules (IDF1–IDF6). The IFCT analysis demonstrated that the engineered molecules show maximum intrinsic charge transfer (62.437% to 81.374%) and minimum intrinsic local excitation (18.626% to 37.563%) than the reference molecule (ICT = 51.471% and ILE = 48.529%), resulting low charge losses with high charge-density flow. Moreover, the high hole hopping rate, high total amount of charge transfer, and low reorganization energy with comparable charge transfer integral demonstrated that designed molecules have effective hole transport ability for photovoltaic cells. The synthetic pathway also indicates that engineered molecules (IDF1–IDF6) can be synthesized experimentally. Our remarkable results demonstrated that IDF1–IDF6 are advantageous molecules for the manufacturing of highly efficient PSCs and OSCs, which may have future commercial applications in the solar industry.

## Conflicts of interest

The authors declare no conflict of interest.

## Supplementary Material

RA-014-D3RA08639A-s001
