# Supplementary material for: Exploring the potential of end-capping acceptor engineering on indolo[3,2-b]indole-based small molecules for efficient organic and perovskite solar cells
Source: RSC Adv. 2024 Feb 9;14(8):5248–63. doi: 10.1039/d3ra08639a (PMC10854478; doi:10.1039/d3ra08639a)
Supplement: RA-014-D3RA08639A-s001 [file RA-014-D3RA08639A-s001.pdf]

Electronic Supplementary Material (ESI) for RSC Advances.
This journal is © The Royal Society of Chemistry 2024

"Exploring the Potential of End-capping Acceptor Engineering on indolo[3,2-b]indole-based small molecules for efficient Organic and Perovskite Solar Cells"

Waqar Ali Zahid[a], Muhammad Fiaz Ahmad[b], Waqas Akram[a], Rabia Iftikhar[a], Javed Iqbal[a]*

[a] *Department of Chemistry, University of Agriculture, Faisalabad-38000, Pakistan.*

[b] *Department of Chemistry, University of Education, Lahore, Pakistan.*

[*]Corresponding author: Email: javedkhattak79@gmail.com; Javed.iqbal@uaf.edu.pk (J.I)

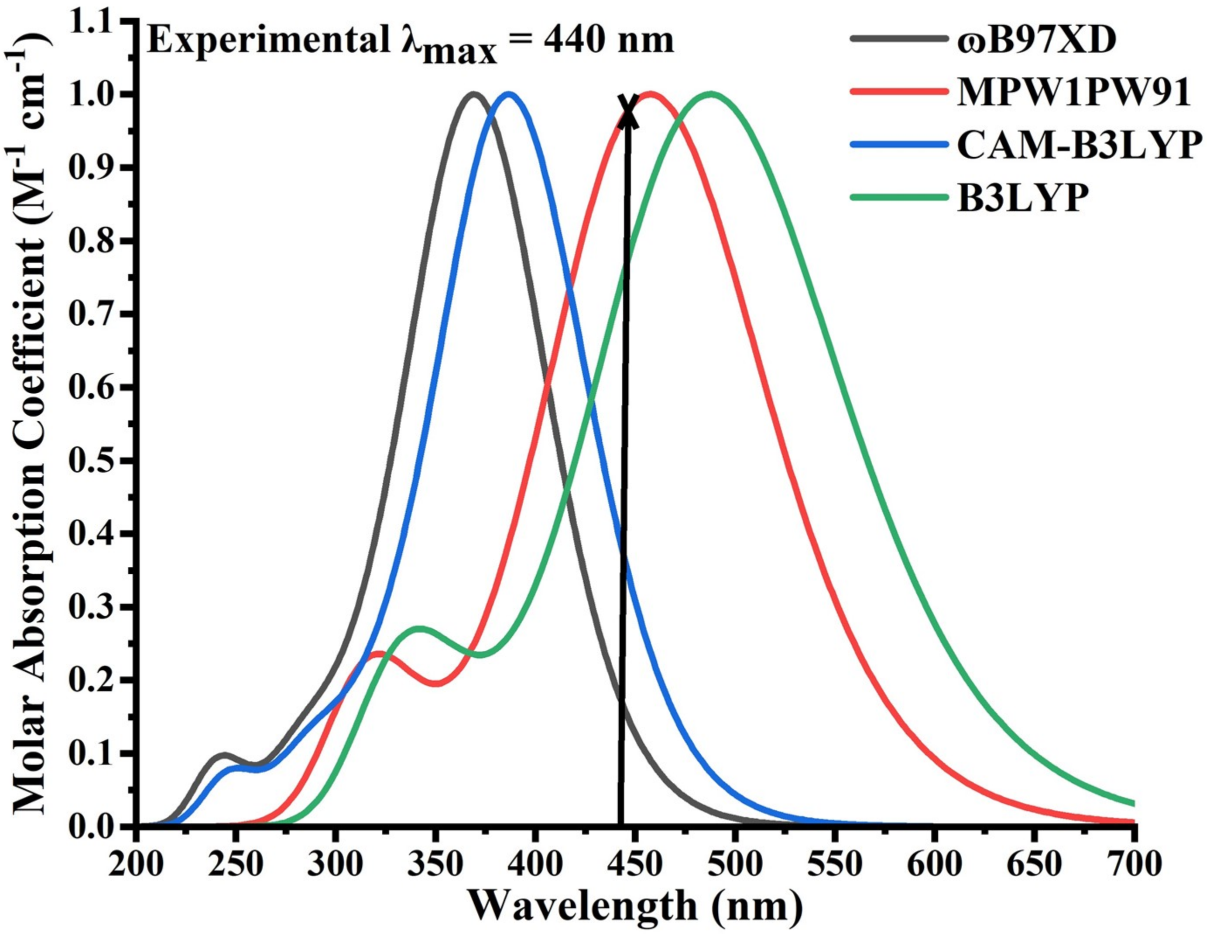

Figure S1. Normalized UV-Vis spectra of four different DFT functionals at "6-31G (d, p)" basis set for the reference (**IDFR**) molecule in solvent (chlorobenzene) phase.

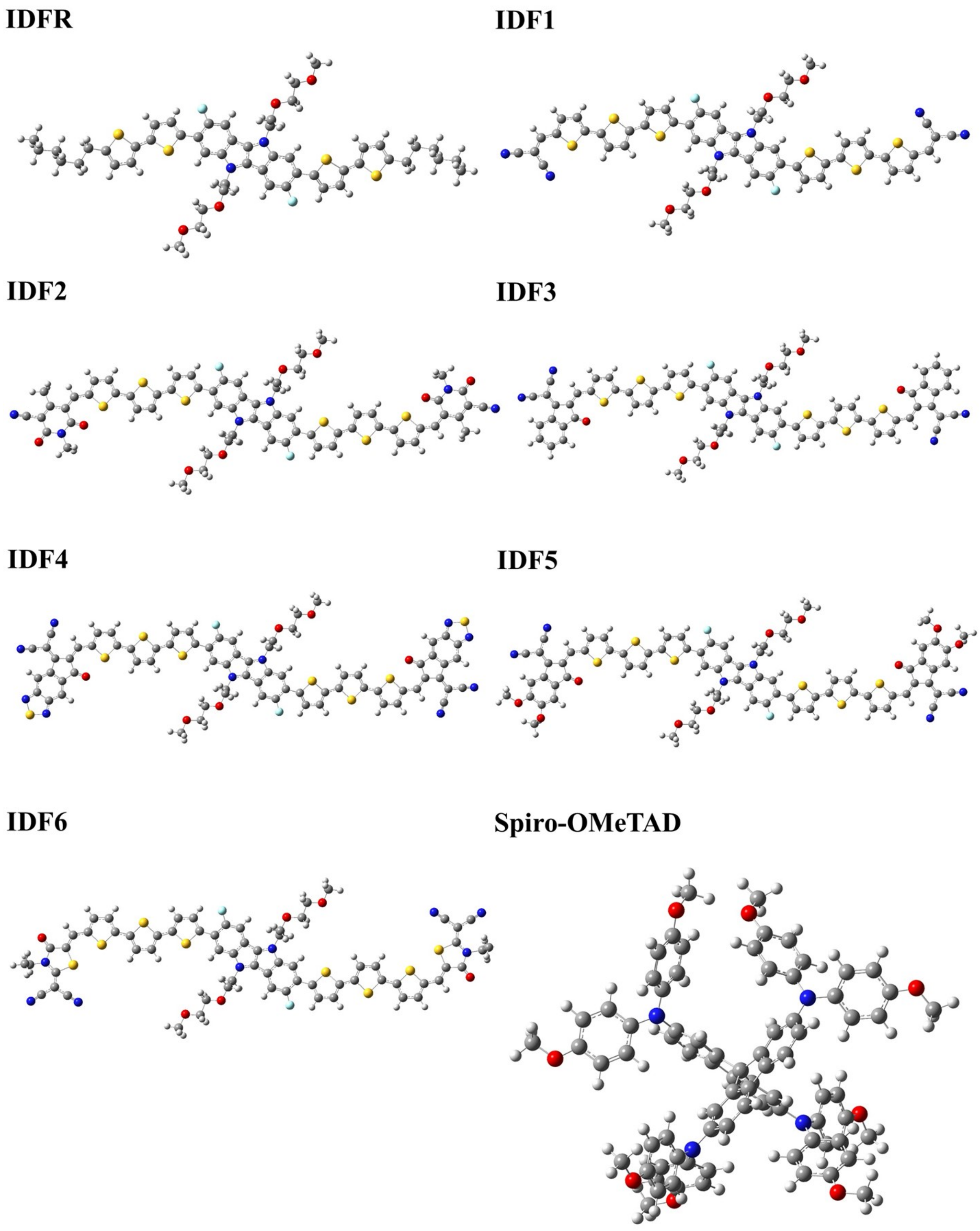

Figure S2. Optimized geometries of **IDFR**, **IDF1**-**IDF6**, and **Spiro-OMeTAD** molecules.

Table S1. Charge transfer length (D_{CT}), electronic coupling parameter (H index), Charge separation parameter (t index), maximum intrinsic charge transfer (CT) and minimum intrinsic local excitation (LE) of investigated molecules.

Molecules	D_{CT} (Å)	H Index	t index	CT (%)	LE (%)
**IDFR**	0.026577	6.560	-0.841	51.471	48.529
**IDF1**	0.060308	10.644	-8.114	66.771	33.229
**IDF2**	0.212426	11.780	-11.075	69.446	30.554
**IDF3**	0.203775	12.685	-12.031	76.754	23.246
**IDF4**	0.104316	13.136	-8.811	81.374	18.626
**IDF5**	14.068894	12.427	-8.128	75.714	24.286
**IDF6**	1.551828	16.218	-13.608	62.437	37.563
**Spiro-OMeTAD**	1.690645	4.951	-1.613	66.235	33.765

Table S2. Cartesian coordinates of the optimized structure of **IDFR** (reference) molecule at the "MPW1PW91/6-31G (d,p)" level of theory.

C	-3.17127	2.61978	-0.4553
C	-3.8647	1.42105	-0.79487
C	-3.2517	0.16175	-0.61146
C	-1.96814	0.15419	-0.08969
C	-1.316	1.32989	0.2331
C	-1.89737	2.57933	0.05071
H	-3.75552	-0.74804	-0.86318
H	-1.37093	3.476	0.29861
C	1.91983	-2.04042	1.29183
C	3.19763	-2.06994	1.79538
C	3.86438	-0.86103	2.16963
C	3.23697	0.38571	1.99294
C	1.9512	0.38068	1.48764
H	1.41005	-2.9404	1.01893
H	3.73213	1.29943	2.24246
N	-1.07307	-0.9869	0.2287
N	1.03575	1.51317	1.22409
C	-0.06903	0.9477	0.72562
C	0.04949	-0.41956	0.68872
C	1.31128	-0.79766	1.1494
C	-7.70504	1.10688	-1.97435
C	-7.36154	2.42412	-1.94019
S	-6.27498	0.09214	-1.79396
C	-5.99525	2.64505	-1.59489
H	-8.05204	3.21579	-2.14139
C	-5.29666	1.49361	-1.36202
H	-5.56226	3.61874	-1.51238
C	7.63818	-0.44912	3.55864
C	7.33167	-1.77408	3.48408
C	5.99937	-2.03099	3.04151
C	5.27902	-0.89949	2.78518

S	6.19803	0.529	3.26538
H	8.02905	-2.54809	3.72799
H	5.60696	-3.01755	2.91352
C	8.92014	0.06046	3.82921
C	9.25421	1.42967	3.85323
S	10.3408	-0.92284	4.20249
C	10.56676	1.67864	4.3398
H	8.5826	2.20365	3.54971
C	11.25438	0.50403	4.7066
H	10.98372	2.65837	4.43104
C	-9.0072	0.61255	-2.1227
C	-9.33017	-0.75581	-2.16371
C	-10.68476	-0.99999	-2.50116
C	-11.42319	0.1773	-2.7204
S	-10.46476	1.5983	-2.29147
H	-8.62206	-1.53271	-1.97023
H	-11.1011	-1.98173	-2.5919
C	1.31173	2.9306	1.47907
C	0.89154	3.24914	2.92558
C	2.60128	4.80971	3.13272
C	2.94043	6.25122	3.55477
C	4.64786	7.78419	3.9257
O	1.18869	4.60976	3.23228
O	4.35109	6.45975	3.47583
H	2.35707	3.11962	1.35982
H	0.76121	3.54302	0.79427
H	1.42927	2.61398	3.59969
H	-0.15863	3.07793	3.03472
H	3.10442	4.12232	3.78008
H	2.91698	4.64315	2.12382
H	2.61611	6.40822	4.56081
H	2.44178	6.94083	2.90656
H	5.7026	7.95151	3.87085
H	4.14079	8.4943	3.30631
H	4.32048	7.89751	4.93876
F	3.82792	-3.25528	1.93778
F	-3.77929	3.81152	-0.63336
C	-1.38466	-2.41109	0.0702
C	-2.0694	-2.90194	1.35988
C	-3.34684	-4.37902	0.11973
C	-3.83871	-5.83284	-0.01977
C	-5.1986	-7.26601	-1.29248
O	-2.44245	-4.27346	1.22098
O	-4.72668	-5.92332	-1.1398
H	-2.04719	-2.5417	-0.75895
H	-0.48701	-2.96803	-0.10384
H	-2.94906	-2.31752	1.53658
H	-1.39615	-2.79313	2.18255
H	-4.18587	-3.73641	0.28665
H	-2.84446	-4.08426	-0.77741
H	-4.35318	-6.12364	0.87209

H	-3.0031	-6.48211	-0.17504
H	-5.83388	-7.32202	-2.15101
H	-4.36559	-7.92456	-1.42077
H	-5.74928	-7.55375	-0.42178
C	-12.86257	0.20419	-3.26702
C	-13.05992	-0.99091	-4.22061
C	-15.4891	-1.1164	-3.59688
C	-16.93228	-1.10157	-4.13308
H	-13.55519	0.13593	-2.45416
H	-13.02913	1.11611	-3.8001
H	-12.88462	-1.90304	-3.69063
H	-12.36936	-0.91121	-5.03342
H	-15.30936	-2.03645	-3.07939
H	-15.35276	-0.29689	-2.92291
H	-17.07525	-1.9246	-4.80286
H	-17.10939	-0.18439	-4.65371
C	-14.49888	-0.98546	-4.76828
C	-17.91605	-1.2203	-2.95398
H	-14.63021	-1.80873	-5.44047
H	-14.67912	-0.06907	-5.28997
H	-17.74196	-2.13947	-2.43461
H	-17.76873	-0.39933	-2.28314
H	-18.91929	-1.2037	-3.32334
C	12.60191	0.47233	5.45439
C	13.61035	1.63401	5.34593
C	15.49697	0.00094	5.06878
C	16.6998	-0.41938	4.1987
H	12.91569	-0.44202	5.91387
H	12.9772	0.16773	4.49812
H	13.98453	1.87126	6.32126
H	13.12022	2.49059	4.93345
H	15.8426	0.25943	6.04636
H	14.80373	-0.81249	5.13663
H	17.39272	0.39289	4.12982
H	16.35864	-0.68183	3.21981
C	14.78866	1.2212	4.44272
C	17.40062	-1.63501	4.83841
H	15.48028	2.03473	4.3657
H	14.42828	0.96916	3.46748
H	17.74537	-1.37336	5.81596
H	16.70859	-2.44848	4.90976
H	18.23293	-1.9281	4.23272

Table S3. Cartesian coordinates of the optimized structure of **IDF1** molecule at the "MPW1PW91/6-31G (d,p)" level of theory.

C	-3.02278	2.79013	-0.58456
C	-3.93969	1.67571	-0.61434
C	-3.44016	0.36989	-0.54097

C	-2.07258	0.19408	-0.47829
C	-1.19289	1.25945	-0.4434
C	-1.65009	2.58214	-0.49909
H	-4.10099	-0.46761	-0.54992
H	-0.96391	3.40581	-0.46911
C	1.72316	-2.59343	-0.22083
C	3.09259	-2.80431	-0.11141
C	4.00965	-1.69519	-0.0801
C	3.51422	-0.38336	-0.15669
C	2.14658	-0.20422	-0.234
H	1.03716	-3.41582	-0.25687
H	4.17727	0.45319	-0.15018
N	-1.31462	-1.07001	-0.42128
N	1.38313	1.06006	-0.26695
C	0.09045	0.68318	-0.36206
C	-0.01759	-0.69554	-0.36031
C	1.26634	-1.27062	-0.27038
C	-8.05235	1.58595	-0.5788
C	-7.60808	2.83059	-0.98149
S	-6.64764	0.50103	-0.48808
C	-6.17089	2.96736	-1.04134
H	-8.2694	3.63243	-1.22659
C	-5.4913	1.82911	-0.72813
H	-5.67698	3.8813	-1.30388
C	8.11271	-1.57184	0.03193
C	7.67718	-2.83464	0.40111
C	6.23806	-2.99091	0.39752
C	5.5561	-1.85458	0.06448
S	6.69945	-0.52242	-0.1622
H	8.33965	-3.6305	0.67021
H	5.74441	-3.91171	0.63885
C	9.46678	-1.10899	-0.18246
C	9.77858	0.23178	-0.50781
S	10.99083	-2.02455	-0.08729
C	11.18804	0.54438	-0.52302
H	9.0197	0.95586	-0.71759
C	12.02773	-0.56766	-0.25935
H	11.55894	1.52579	-0.72206
C	-9.42258	1.19504	-0.28746
C	-9.82856	-0.13752	-0.03341
C	-11.23213	-0.29567	0.29393
C	-11.9693	0.92895	0.34664
S	-10.87296	2.2408	-0.202
H	-9.14164	-0.96044	-0.07078
H	-11.66841	-1.2548	0.48434
C	1.96101	2.41298	-0.19328
C	1.96626	2.84435	1.29137
C	3.92138	4.21933	1.33379
C	4.3855	5.67661	1.55028
C	6.24146	7.09093	1.7437
O	2.49399	4.17044	1.45518

O	5.81421	5.7436	1.50591
H	2.96009	2.40447	-0.57989
H	1.36765	3.09396	-0.76287
H	2.55968	2.15988	1.86306
H	0.95801	2.83048	1.64234
H	4.36001	3.5912	2.08228
H	4.22474	3.88085	0.36554
H	4.04798	6.0164	2.50763
H	3.97222	6.2995	0.78435
H	7.31072	7.1333	1.72934
H	5.84856	7.72958	0.98098
H	5.88537	7.41704	2.69975
F	3.5647	-4.06822	-0.04079
F	-3.49682	4.0537	-0.64299
C	17.70373	-0.53988	-0.25479
C	18.66734	0.41292	-0.48276
H	18.11356	-1.51559	-0.13926
C	18.49835	1.7704	-0.76001
C	19.99503	-0.05998	-0.42447
N	21.06656	-0.46814	-0.37284
N	18.44226	2.8968	-0.99491
C	-17.54969	0.78325	0.65813
C	-18.42202	-0.2865	0.58256
H	-18.03849	1.71297	0.51407
C	-18.16428	-1.6575	0.38928
C	-19.77181	0.05961	0.71957
N	-20.87731	0.34304	0.83179
N	-18.05812	-2.7922	0.23131
C	-1.91321	-2.41532	-0.43294
C	-2.14146	-2.83622	1.03372
C	-4.12226	-4.14295	0.8118
C	-4.66221	-5.5843	0.93567
C	-6.57729	-6.9296	0.83941
O	-2.72224	-4.14619	1.12225
O	-6.07363	-5.59531	0.69878
H	-2.84606	-2.3954	-0.96042
H	-1.25351	-3.10727	-0.9104
H	-2.79443	-2.13395	1.51094
H	-1.19445	-2.84323	1.52521
H	-4.63485	-3.50984	1.50767
H	-4.2822	-3.77994	-0.18142
H	-4.46872	-5.95148	1.92242
H	-4.17231	-6.21045	0.21941
H	-7.63569	-6.92937	0.68059
H	-6.10824	-7.56537	0.11847
H	-6.36561	-7.29151	1.8249
C	13.61471	-0.6165	-0.12151
C	14.20942	-1.81987	-0.03178
C	15.74187	-1.7628	-0.0576
C	16.26702	-0.53499	-0.15272
S	14.91257	0.6723	-0.09268

H	13.66679	-2.73249	0.04667
H	16.35622	-2.63667	-0.03368
C	-13.50335	1.10405	0.78379
C	-14.11521	2.30971	1.05552
C	-15.66542	2.17938	1.1104
C	-16.13921	0.91946	0.88762
S	-14.74705	-0.20928	0.98506
H	-13.6053	3.24824	1.19638
H	-16.31671	3.01347	1.30962

Table S4. Cartesian coordinates of the optimized structure of **IDF2** molecule at the "MPW1PW91/6-31G (d,p)" level of theory.

C	2.99238	-2.82686	-0.57621
C	3.8959	-1.72948	-0.64727
C	3.42695	-0.41455	-0.61068
C	2.06871	-0.229	-0.49299
C	1.1813	-1.28529	-0.40777
C	1.63205	-2.61452	-0.44543
H	4.09819	0.41562	-0.67366
H	0.95551	-3.43855	-0.37039
C	-1.69327	2.60731	-0.14232
C	-3.05208	2.82367	-0.00169
C	-3.95738	1.7285	0.0706
C	-3.48967	0.41151	0.02217
C	-2.13221	0.22201	-0.09946
H	-1.01465	3.42981	-0.2083
H	-4.16185	-0.41976	0.09003
N	1.32957	1.04127	-0.43595
N	-1.39203	-1.04901	-0.15239
C	-0.09548	-0.69253	-0.28673
C	0.03209	0.68503	-0.30826
C	-1.24436	1.27748	-0.18584
C	7.92344	-1.65057	-0.71412
C	7.50419	-2.89685	-1.12203
S	6.54103	-0.64614	-0.31151
C	6.07967	-3.05344	-1.13259
H	8.1754	-3.67427	-1.41707
C	5.41601	-1.92694	-0.74357
H	5.58745	-3.95984	-1.42547
C	-7.99707	1.64631	0.21735
C	-7.56128	2.88784	0.63064
C	-6.13409	3.04597	0.60308
C	-5.47724	1.91779	0.20827
S	-6.61196	0.61136	-0.11559
H	-8.22243	3.66832	0.93606
H	-5.63418	3.95326	0.88474
C	-9.35246	1.22485	0.06042
C	-9.7446	-0.06802	-0.16997

S	-10.78408	2.26177	0.12898
C	-11.18258	-0.25318	-0.25629
H	-9.0403	-0.87739	-0.26014
C	-11.92299	0.90597	-0.11076
H	-11.6212	-1.20981	-0.42528
C	9.26541	-1.2131	-0.6102
C	9.65591	0.07295	-0.34488
C	11.08196	0.24082	-0.23254
C	11.79846	-0.91641	-0.42462
S	10.68029	-2.26101	-0.71812
H	8.95853	0.88073	-0.22625
H	11.53663	1.18784	-0.03506
C	-2.00132	-2.3829	-0.06148
C	-1.98891	-2.80856	1.42304
C	-3.94282	-4.18301	1.5293
C	-4.37294	-5.63706	1.77799
C	-6.24874	-7.03582	2.02479
O	-2.51693	-4.12839	1.59634
O	-5.79969	-5.70125	1.77308
H	-3.00743	-2.35242	-0.43178
H	-1.4324	-3.07541	-0.64008
H	-2.57202	-2.11857	2.00074
H	-0.97831	-2.80391	1.76023
H	-4.35728	-3.55172	2.28977
H	-4.28984	-3.85778	0.57084
H	-4.00193	-5.9626	2.7293
H	-3.97657	-6.2668	1.00742
H	-7.31759	-7.04933	2.01885
H	-5.87482	-7.68745	1.26235
H	-5.88999	-7.36202	2.98067
F	-3.5327	4.08115	0.06417
F	3.47664	-4.08574	-0.62492
C	1.94694	2.37321	-0.49978
C	2.26912	2.81724	0.94356
C	4.20897	4.16708	0.58047
C	4.70195	5.61771	0.70097
C	6.59819	6.99327	0.47552
O	2.83512	4.13286	0.9743
O	6.08951	5.66086	0.36434
H	2.84615	2.33202	-1.08287
H	1.2659	3.06154	-0.94791
H	2.96127	2.12766	1.38588
H	1.35998	2.82794	1.49865
H	4.78047	3.53905	1.23444
H	4.32225	3.82562	-0.42729
H	4.56542	5.96036	1.70727
H	4.14515	6.24371	0.03363
H	7.63676	6.99073	0.22229
H	6.06447	7.6353	-0.1944
H	6.47377	7.34357	1.48075
C	-13.37697	1.10129	-0.13371

C	-13.9093	2.35152	0.21415
C	-15.30411	2.4837	0.05422
C	-16.00947	1.41265	-0.48044
S	-14.79397	0.01444	-0.56046
H	-13.30275	3.16292	0.55006
H	-15.82948	3.36681	0.33554
C	13.22005	-1.08706	-0.39754
C	13.81849	-2.34063	-0.63411
C	15.23017	-2.32228	-0.64565
C	15.81627	-1.07509	-0.45966
S	14.5125	0.11716	-0.0565
H	13.25695	-3.2303	-0.8113
H	15.82525	-3.19847	-0.7843
C	17.25003	-0.97954	-0.62318
C	18.04226	0.10943	-0.43128
C	17.44784	1.47866	-0.12797
C	19.5862	-0.0069	-0.52312
C	20.35352	1.09884	-0.38339
C	19.69328	2.46418	-0.06351
O	20.36351	3.51624	-0.20567
O	16.22612	1.67783	-0.32631
C	20.2746	-1.37003	-0.79625
C	21.75489	1.03223	-0.46715
H	19.85954	-2.11607	-0.14671
H	20.10483	-1.65287	-1.81249
H	21.32493	-1.28047	-0.61673
H	17.76613	-1.88536	-0.90587
N	22.90303	0.97766	-0.53578
N	18.30538	2.53761	0.43066
C	17.6908	3.82676	0.12119
H	16.8242	3.93339	0.72524
H	18.39102	4.61028	0.34009
H	17.41877	3.88543	-0.90927
C	-17.43875	1.79794	-0.77527
C	-18.65888	1.19511	-1.21951
C	-18.83321	-0.222	-1.79135
C	-19.99663	2.05438	-1.12923
C	-21.19647	1.54504	-1.54918
C	-21.2845	0.10014	-2.05886
O	-22.32305	-0.27166	-2.67754
O	-17.83631	-0.85325	-2.19902
C	-20.01055	3.50562	-0.59381
C	-22.39654	2.29139	-1.44835
H	-19.45802	3.55603	0.32153
H	-19.56667	4.15405	-1.31478
H	-21.02647	3.80737	-0.41782
H	-17.59417	2.74888	-0.58854
N	-23.40196	2.86134	-1.40377
N	-20.18994	-0.86017	-1.81519
C	-20.18919	-1.9151	-2.8266
H	-19.5915	-2.71363	-2.46813

H	-21.19813	-2.25148	-2.98516
H	-19.79707	-1.55963	-3.75353

Table S5. Cartesian coordinates of the optimized structure of **IDF3** molecule at the "MPW1PW91/6-31G (d,p)" level of theory.

C	2.96557	-2.82888	-0.60494
C	3.89644	-1.72164	-0.6609
C	3.42064	-0.41161	-0.56854
C	2.06279	-0.21768	-0.47765
C	1.17244	-1.27549	-0.42148
C	1.60399	-2.60252	-0.47793
H	4.09464	0.41446	-0.57206
H	0.90508	-3.41748	-0.4246
C	-1.66901	2.59331	-0.12324
C	-3.02962	2.81913	0.0261
C	-3.96407	1.71216	0.08465
C	-3.48647	0.40163	-0.01783
C	-2.12732	0.20824	-0.12323
H	-0.9707	3.40926	-0.17805
H	-4.15853	-0.4255	-0.00467
N	1.3189	1.05626	-0.41965
N	-1.37799	-1.06526	-0.16388
C	-0.0923	-0.69238	-0.30098
C	0.02898	0.68493	-0.31112
C	-1.23728	1.26732	-0.19118
C	7.97546	-1.61458	-0.81643
C	7.54065	-2.88095	-1.11873
S	6.58486	-0.52193	-0.76136
C	6.11216	-3.0322	-1.08812
H	8.20292	-3.68526	-1.34946
C	5.43765	-1.87618	-0.83948
H	5.61826	-3.96674	-1.24143
C	-8.06915	1.63112	0.28247
C	-7.60996	2.89253	0.58974
C	-6.17393	3.03065	0.54911
C	-5.5088	1.8731	0.28054
S	-6.67628	0.53429	0.19744
H	-8.25757	3.70413	0.82928
H	-5.66915	3.95978	0.71098
C	-9.43591	1.23288	0.04705
C	-9.81636	-0.05089	-0.22644
S	-10.88202	2.25993	0.04545
C	-11.24268	-0.26991	-0.32069
H	-9.09988	-0.82982	-0.35207
C	-11.99236	0.87213	-0.13919
H	-11.66515	-1.23808	-0.5084
C	9.3224	-1.22065	-0.57122
C	9.71686	0.05625	-0.28607

C	11.14192	0.24814	-0.21394
C	11.85042	-0.91265	-0.4051
S	10.73082	-2.27846	-0.57316
H	9.01506	0.84426	-0.13693
H	11.59202	1.20451	-0.0278
C	-1.95264	-2.42392	-0.04589
C	-1.92248	-2.82784	1.45163
C	-3.90291	-4.17796	1.51688
C	-4.41024	-5.62792	1.7682
C	-6.25072	-7.0352	2.00694
O	-2.46932	-4.14827	1.65101
O	-5.84149	-5.68583	1.72145
H	-2.96165	-2.42738	-0.40666
H	-1.37168	-3.11581	-0.61755
H	-2.49123	-2.12486	2.02622
H	-0.90315	-2.81873	1.77765
H	-4.33471	-3.51866	2.24257
H	-4.18553	-3.86011	0.53563
H	-4.08841	-5.94911	2.73618
H	-4.00717	-6.27711	1.01967
H	-7.3195	-7.09547	2.01443
H	-5.8626	-7.69174	1.25631
H	-5.87214	-7.32741	2.96576
F	-3.46967	4.091	0.12143
F	3.41005	-4.10133	-0.67369
C	1.91208	2.40962	-0.46103
C	2.20929	2.84202	0.99004
C	4.16475	4.16307	0.57838
C	4.73325	5.60838	0.68194
C	6.59519	6.99172	0.45896
O	2.79921	4.15736	1.03744
O	6.11552	5.64405	0.30419
H	2.82051	2.39441	-1.0309
H	1.22592	3.09509	-0.91426
H	2.88374	2.14178	1.44065
H	1.28614	2.8517	1.5301
H	4.74377	3.50702	1.19671
H	4.21102	3.82744	-0.43615
H	4.6478	5.94734	1.69273
H	4.17539	6.25334	0.0363
H	7.63734	7.03498	0.21785
H	6.05147	7.64094	-0.19534
H	6.45221	7.30707	1.47288
C	-13.42928	1.02498	-0.10194
C	-13.98737	2.27633	0.18052
C	-15.38664	2.35249	-0.03088
C	-16.01478	1.14691	-0.42123
S	-14.77195	-0.15523	-0.38685
H	-13.40204	3.10952	0.51971
H	-15.9298	3.26108	0.08952
C	13.25396	-1.11828	-0.45914

C	13.73667	-2.42095	-0.56857
C	15.14454	-2.5223	-0.53835
C	15.8253	-1.29097	-0.45849
S	14.62469	0.03445	-0.34362
H	13.08578	-3.26856	-0.66569
H	15.64985	-3.4572	-0.58226
C	17.25712	-1.25103	-0.49196
C	18.03315	-0.14708	-0.70854
C	17.63031	1.29988	-1.05183
C	19.57455	-0.1531	-0.67313
C	18.94346	2.08523	-1.29882
C	20.0409	1.26507	-1.06854
C	21.34508	1.73677	-1.19911
C	21.5303	3.07415	-1.60277
C	20.41518	3.91066	-1.85555
C	19.10163	3.41525	-1.69991
H	22.184	1.10502	-1.00169
H	22.52103	3.46043	-1.71818
H	20.57301	4.92319	-2.16732
H	18.25278	4.04393	-1.88526
H	17.77968	-2.17921	-0.33996
O	16.46487	1.76433	-1.12812
C	20.38283	-1.20592	-0.35618
C	21.77529	-1.04315	-0.38313
C	19.8439	-2.45437	0.00367
N	22.91551	-0.91987	-0.40245
N	19.41385	-3.47921	0.29916
C	-17.43035	1.18707	-0.74666
C	-18.33552	0.17938	-1.05022
C	-18.19445	-1.3509	-1.20451
C	-19.8355	0.46444	-1.29969
C	-19.62768	-1.92613	-1.48166
C	-20.54456	-0.88467	-1.53122
C	-21.90522	-1.10251	-1.75774
C	-22.35211	-2.4247	-1.92805
C	-21.43484	-3.50144	-1.88129
C	-20.05181	-3.25988	-1.664
H	-22.59439	-0.2862	-1.80143
H	-23.39256	-2.61501	-2.0924
H	-21.79599	-4.50335	-2.01184
H	-19.35663	-4.07612	-1.63899
H	-17.86398	2.16576	-0.74221
O	-17.1319	-2.00709	-1.11747
C	-20.44532	1.68227	-1.31032
C	-21.83209	1.75126	-1.51459
C	-19.71459	2.86816	-1.12146
N	-22.96544	1.81296	-1.68112
N	-19.12987	3.84574	-0.96786

Table S6. Cartesian coordinates of the optimized structure of **IDF4** molecule at the "MPW1PW91/6-31G (d,p)" level of theory.

C	-2.88067	2.94029	-0.47093
C	-3.83341	1.87221	-0.59665
C	-3.41109	0.53884	-0.54245
C	-2.06053	0.29717	-0.44249
C	-1.14258	1.32452	-0.33879
C	-1.5336	2.66714	-0.34164
H	-4.11808	-0.26476	-0.57804
H	-0.81473	3.45517	-0.24646
C	1.58287	-2.63911	-0.17595
C	2.92752	-2.91393	-0.02471
C	3.87994	-1.84611	0.10619
C	3.46053	-0.51233	0.03719
C	2.11026	-0.2682	-0.06738
H	0.86486	-3.42722	-0.28109
H	4.16913	0.28968	0.07094
N	-1.35204	-0.99711	-0.41723
N	1.39831	1.02656	-0.07217
C	0.10443	0.69904	-0.23023
C	-0.05477	-0.67043	-0.28764
C	1.19265	-1.29585	-0.18257
C	-7.86981	1.92356	-0.79503
C	-7.40575	3.1964	-1.01463
S	-6.52582	0.78177	-0.80676
C	-5.97581	3.29695	-0.99924
H	-8.04888	4.03765	-1.16971
C	-5.34865	2.1022	-0.79903
H	-5.44931	4.21876	-1.12253
C	7.91244	-1.90236	0.36867
C	7.4435	-3.17377	0.58359
C	6.01403	-3.27253	0.54765
C	5.39129	-2.07707	0.33386
S	6.57024	-0.75829	0.35575
H	8.08356	-4.01575	0.74664
H	5.48467	-4.19352	0.66888
C	9.26237	-1.54148	0.16544
C	9.70821	-0.26101	-0.01896
S	10.61541	-2.66656	0.10201
C	11.13269	-0.14327	-0.07901
H	9.0486	0.57684	-0.10481
C	11.78138	-1.34205	0.01855
H	11.63768	0.7958	-0.18378
C	-9.21702	1.56013	-0.57423
C	-9.65804	0.27922	-0.38089
C	-11.08219	0.15801	-0.30644
C	-11.73606	1.35368	-0.40459

S	-10.57312	2.68033	-0.50467
H	-8.99593	-0.55713	-0.30192
H	-11.58335	-0.78172	-0.19258
C	2.0143	2.35599	0.08484
C	1.99336	2.72856	1.58857
C	4.00183	4.03948	1.6958
C	4.50396	5.46803	2.00083
C	6.37664	6.83513	2.27565
O	2.56965	4.02617	1.81753
O	5.93365	5.51147	1.94771
H	3.02141	2.32915	-0.26992
H	1.45854	3.08069	-0.46914
H	2.54498	2.00196	2.14942
H	0.97517	2.74	1.91338
H	4.42578	3.35466	2.40356
H	4.29379	3.75251	0.70782
H	4.18258	5.74975	2.98239
H	4.09924	6.14872	1.28104
H	7.44689	6.86608	2.27265
H	5.99818	7.52857	1.55327
H	6.01548	7.10066	3.24838
F	3.34295	-4.19854	-0.00131
F	-3.29875	4.22362	-0.47324
C	-1.98122	-2.32484	-0.50635
C	-2.295	-2.8145	0.92134
C	-4.26779	-4.10275	0.45943
C	-4.82312	-5.54051	0.55135
C	-6.70148	-6.90221	0.29623
O	-2.90301	-4.11683	0.91095
O	-6.20039	-5.56535	0.1635
H	-2.88455	-2.25498	-1.07322
H	-1.31262	-3.00897	-0.98472
H	-2.9621	-2.12612	1.39744
H	-1.37667	-2.86515	1.46401
H	-4.84615	-3.45661	1.08979
H	-4.32256	-3.75235	-0.55009
H	-4.73901	-5.8834	1.56202
H	-4.25921	-6.18142	-0.0938
H	-7.74123	-6.92221	0.04166
H	-6.16148	-7.55359	-0.35958
H	-6.5762	-7.23076	1.30794
C	13.17315	-1.54565	0.03384
C	13.74373	-2.82207	0.16933
C	15.15109	-2.82127	0.07763
C	15.71948	-1.54421	-0.08558
S	14.44151	-0.31318	-0.18606
H	13.16556	-3.71223	0.32806
H	15.73556	-3.71413	0.13419
C	-13.13065	1.55246	-0.41122
C	-13.70854	2.82609	-0.54421
C	-15.11617	2.8198	-0.4434

C	-15.67857	1.54102	-0.27532
S	-14.39381	0.31659	-0.17945
H	-13.13543	3.71823	-0.70642
H	-15.70451	3.71018	-0.49781
C	-17.07974	1.37793	-0.21607
C	-17.73775	0.19102	-0.05227
C	-17.18005	-1.23783	0.09999
C	-19.2671	0.05131	0.01113
C	-18.407	-2.19323	0.19384
C	-19.59243	-1.45087	0.14655
C	-20.85412	-2.04241	0.19888
C	-20.88349	-3.44712	0.26482
C	-19.66915	-4.19091	0.30998
C	-18.41163	-3.54168	0.29087
H	-21.74364	-1.44111	0.17718
H	-17.49333	-4.08517	0.34006
H	-17.67024	2.26549	-0.30749
O	-15.96265	-1.56148	0.13627
C	-20.15461	1.08015	-0.05082
C	-21.52595	0.8353	0.03527
C	-19.68063	2.40017	-0.20443
N	-22.65208	0.67272	0.10127
N	-19.30893	3.47789	-0.32847
C	17.12136	-1.3868	-0.13756
C	17.78664	-0.20328	-0.29399
C	17.24049	1.22882	-0.45032
C	19.31586	-0.07263	-0.34225
C	18.4731	2.17729	-0.52264
C	19.65436	1.42483	-0.46845
C	20.92104	2.00617	-0.50539
C	20.95744	3.39994	-0.55467
C	19.75401	4.15899	-0.601
C	18.48872	3.52848	-0.60569
H	21.81394	1.41089	-0.48295
H	17.57694	4.08889	-0.65955
H	17.70633	-2.27703	-0.04494
O	16.02779	1.5619	-0.50293
C	20.19378	-1.10455	-0.2747
C	21.56439	-0.86239	-0.35047
C	19.71948	-2.42256	-0.12511
N	22.68726	-0.6817	-0.41091
N	19.35272	-3.50383	-0.00317
N	19.93949	5.44096	-0.6238
N	22.04566	4.09696	-0.54799
N	-21.9644	-4.1651	0.28198
N	-19.83434	-5.4764	0.36052
S	21.58631	5.71209	-0.30439
S	-21.48511	-5.78362	0.1005

Table S7. Cartesian coordinates of the optimized structure of **IDF5** molecule at the "MPW1PW91/6-31G (d,p)" level of theory.

C	-2.88643	2.93323	-0.47999
C	-3.83673	1.86457	-0.60876
C	-3.41191	0.52998	-0.54904
C	-2.06242	0.29087	-0.4362
C	-1.14659	1.32058	-0.3285
C	-1.53798	2.66093	-0.3403
H	-4.11358	-0.27555	-0.5954
H	-0.81783	3.44794	-0.24569
C	1.58651	-2.6317	-0.17723
C	2.93293	-2.90609	-0.02001
C	3.8836	-1.83826	0.11548
C	3.46173	-0.50235	0.04358
C	2.11136	-0.26101	-0.06522
H	0.86743	-3.41834	-0.28981
H	4.16536	0.30121	0.08076
N	-1.35159	-1.00251	-0.40725
N	1.39607	1.03218	-0.06715
C	0.10229	0.69952	-0.22172
C	-0.05405	-0.66998	-0.28177
C	1.19525	-1.29118	-0.181
C	-7.86283	1.92106	-0.81479
C	-7.40514	3.19694	-1.00623
S	-6.52315	0.78246	-0.88477
C	-5.97658	3.29971	-0.99772
H	-8.05246	4.03902	-1.134
C	-5.3488	2.09998	-0.82636
H	-5.4533	4.22616	-1.1088
C	7.91222	-1.90095	0.38381
C	7.44592	-3.17508	0.57177
C	6.01645	-3.27464	0.54263
C	5.3935	-2.07461	0.35395
S	6.57106	-0.76027	0.42499
H	8.0879	-4.01831	0.71526
H	5.48911	-4.19956	0.64784
C	9.26157	-1.5398	0.171
C	9.70908	-0.26082	-0.01999
S	10.61577	-2.66822	0.09521
C	11.13388	-0.14708	-0.09486
H	9.05164	0.58152	-0.09673
C	11.78643	-1.34243	0.00323
H	11.64038	0.78696	-0.21308
C	-9.20438	1.55928	-0.57991
C	-9.64954	0.27956	-0.38285
C	-11.07031	0.17145	-0.29241
C	-11.71449	1.36757	-0.39189
S	-10.55021	2.6924	-0.48629
H	-8.99457	-0.56412	-0.31086

H	-11.58164	-0.76029	-0.16488
C	2.00605	2.36677	0.08853
C	1.98287	2.7336	1.59215
C	3.9904	4.0397	1.69091
C	4.50454	5.46559	1.99961
C	6.37571	6.83024	2.26931
O	2.56041	4.02984	1.82642
O	5.93361	5.50598	1.93717
H	3.01491	2.34805	-0.27008
H	1.44742	3.09007	-0.46562
H	2.53144	2.00171	2.14765
H	0.96416	2.74592	1.9152
H	4.41749	3.34851	2.38908
H	4.27018	3.75816	0.69787
H	4.19267	5.74516	2.98398
H	4.09714	6.15058	1.28552
H	7.44563	6.86429	2.26156
H	5.99198	7.52502	1.55197
H	6.01883	7.09123	3.24478
F	3.35154	-4.19069	0.00812
F	-3.30833	4.21654	-0.49352
C	-1.97615	-2.33475	-0.49524
C	-2.28449	-2.82185	0.93429
C	-4.25475	-4.10365	0.4591
C	-4.82348	-5.53878	0.54928
C	-6.69943	-6.89676	0.29063
O	-2.89581	-4.12328	0.92575
O	-6.19817	-5.55903	0.15331
H	-2.88222	-2.27123	-1.06364
H	-1.30703	-3.01857	-0.97384
H	-2.94585	-2.12937	1.41308
H	-1.36408	-2.87596	1.4746
H	-4.83389	-3.45105	1.08037
H	-4.29569	-3.75696	-0.55239
H	-4.7493	-5.88107	1.56031
H	-4.25892	-6.18378	-0.09089
H	-7.73774	-6.91976	0.03136
H	-6.15551	-7.55008	-0.35897
H	-6.57888	-7.22052	1.30436
C	13.1894	-1.53253	0.01446
C	13.77405	-2.79698	0.16343
C	15.18932	-2.78618	0.06603
C	15.75445	-1.49827	-0.12263
S	14.4503	-0.29138	-0.22847
H	13.20237	-3.68811	0.32923
H	15.77684	-3.6782	0.13672
C	-13.10669	1.54629	-0.40719
C	-13.70981	2.80484	-0.53692
C	-15.12184	2.76046	-0.45599
C	-15.64898	1.44935	-0.30563
S	-14.33186	0.27328	-0.19423

H	-13.1543	3.71184	-0.67855
H	-15.73097	3.64016	-0.50768
C	-17.04209	1.21178	-0.26266
C	-17.70656	0.00804	-0.10341
C	-17.26948	-1.50365	0.01788
C	-19.22027	-0.02281	0.00351
C	-18.59852	-2.35018	0.20103
C	-19.65789	-1.45869	0.22553
C	-20.94737	-1.83078	0.44387
C	-21.23702	-3.1513	0.69134
C	-20.22185	-4.15239	0.57163
C	-18.84105	-3.72318	0.32829
H	-21.72575	-1.10078	0.42604
H	-18.03714	-4.4297	0.25354
H	-17.66788	2.07894	-0.36054
O	-16.09554	-1.9545	-0.01544
C	-20.07623	1.02934	-0.07544
C	-21.45721	0.79818	0.01111
C	-19.60753	2.33915	-0.23993
N	-22.58631	0.6084	0.08196
N	-19.24149	3.41541	-0.37408
C	17.16947	-1.3007	-0.19771
C	17.83353	-0.0988	-0.37559
C	17.29769	1.33565	-0.55771
C	19.3719	0.03282	-0.4108
C	18.53737	2.28488	-0.568
C	19.71769	1.54434	-0.5196
C	20.98257	2.17042	-0.54493
C	21.01177	3.57938	-0.3729
C	19.7865	4.30693	-0.35582
C	18.549	3.64216	-0.57841
H	21.88787	1.6046	-0.66225
H	17.64671	4.19709	-0.72903
H	17.78062	-2.17326	-0.09555
O	16.09297	1.68242	-0.66607
C	20.24341	-1.01269	-0.32768
C	21.62655	-0.82162	-0.39086
C	19.74668	-2.31347	-0.165
N	22.76439	-0.691	-0.44096
N	19.34602	-3.37918	-0.03219
O	19.8012	5.72013	-0.05063
O	22.25785	4.28763	-0.16952
O	-22.59817	-3.41517	1.06867
O	-20.69142	-5.53397	0.70117
C	19.86636	6.53362	-1.21546
C	23.05703	4.52671	-1.33777
C	-19.64402	-6.50945	0.82785
C	-23.01381	-4.69767	0.65066
H	20.74948	6.30169	-1.76768
H	19.00523	6.35879	-1.82087
H	19.89133	7.56724	-0.91786

H	23.41729	3.59583	-1.72079
H	22.47884	5.0207	-2.08755
H	23.89031	5.1466	-1.06521
H	-18.97481	-6.23345	1.60973
H	-20.08321	-7.46867	1.05723
H	-19.11325	-6.57879	-0.0955
H	-22.42925	-5.43823	1.14687
H	-24.04846	-4.82829	0.90133
H	-22.88879	-4.78475	-0.40603

Table S8. Cartesian coordinates of the optimized structure of **IDF6** molecule at the "MPW1PW91/6-31G (d,p)" level of theory.

C	3.28396	-2.77209	0.81816
C	4.12964	-1.654	0.55703
C	3.60388	-0.46915	0.03088
C	2.25493	-0.42391	-0.21692
C	1.42372	-1.49488	0.05352
C	1.9243	-2.69522	0.57887
H	4.23321	0.37111	-0.17817
H	1.27971	-3.52204	0.78875
C	-1.59576	1.86734	-1.67119
C	-2.95806	1.95085	-1.89553
C	-3.80727	0.83236	-1.63283
C	-3.2783	-0.35558	-1.11781
C	-1.9264	-0.40663	-0.88305
H	-0.95075	2.69448	-1.87954
H	-3.90756	-1.19633	-0.90849
N	1.4674	0.68934	-0.77248
N	-1.13767	-1.5184	-0.32471
C	0.13431	-1.06378	-0.3105
C	0.19626	0.23171	-0.79702
C	-1.09416	0.66349	-1.15644
C	8.0459	-1.0959	1.2332
C	7.80067	-2.43742	1.2398
S	6.60636	-0.19505	0.79328
C	6.43074	-2.76432	1.01852
H	8.56208	-3.17161	1.40335
C	5.64413	-1.67009	0.80245
H	6.06086	-3.76756	1.01416
C	-7.75788	0.29417	-2.27581
C	-7.49184	1.63868	-2.27499
C	-6.11108	1.955	-2.06715
C	-5.3286	0.85634	-1.86431
S	-6.30944	-0.61064	-1.85243
H	-8.24365	2.38347	-2.42276
H	-5.73107	2.95688	-2.06293
C	-9.00163	-0.30499	-2.56303
C	-9.27151	-1.63561	-2.39808

S	-10.42479	0.53435	-3.17488
C	-10.62295	-1.99966	-2.69479
H	-8.53778	-2.3384	-2.06242
C	-11.39614	-0.9438	-3.08465
H	-10.99309	-3.00033	-2.60799
C	9.26818	-0.50389	1.53249
C	9.53399	0.8277	1.38358
C	10.87791	1.16535	1.66551
C	11.63638	0.10408	2.03306
S	10.67795	-1.37191	2.12791
H	8.80594	1.54653	1.07096
H	11.26387	2.16126	1.60262
C	-1.68267	-2.81293	0.12339
C	-1.99343	-2.69625	1.63219
C	-3.85409	-4.18728	1.81305
C	-4.29144	-5.51801	2.46384
C	-6.09266	-6.97727	2.81183
O	-2.49123	-3.93215	2.16806
O	-5.67307	-5.75992	2.1845
H	-2.57711	-3.04572	-0.42264
H	-0.96206	-3.58323	-0.03862
H	-2.72192	-1.92666	1.78992
H	-1.0876	-2.44671	2.13708
H	-4.46666	-3.39147	2.18152
H	-3.95819	-4.25098	0.75035
H	-4.15189	-5.45772	3.52294
H	-3.69903	-6.31816	2.07176
H	-7.12943	-7.14284	2.6062
H	-5.51765	-7.79246	2.42799
H	-5.94708	-6.90604	3.87014
F	-3.49348	3.09329	-2.37139
F	3.81954	-3.90963	1.30757
C	2.01398	1.99651	-1.18102
C	1.95958	2.9295	0.0448
C	3.86948	4.30255	-0.37951
C	4.28299	5.75848	-0.68938
C	6.10014	7.20741	-1.00142
O	2.44283	4.24519	-0.26812
O	5.70949	5.85491	-0.7365
H	3.02846	1.8843	-1.51641
H	1.42696	2.40209	-1.97671
H	2.55456	2.51613	0.83473
H	0.94307	3.00422	0.35864
H	4.3058	3.99736	0.54835
H	4.2092	3.65503	-1.16029
H	3.91169	6.40423	0.07874
H	3.87095	6.05282	-1.63186
H	7.168	7.27028	-1.0228
H	5.7053	7.51348	-1.94641
H	5.7216	7.84915	-0.23263
C	-12.7742	-0.96651	-3.39371

C	-13.47779	0.20934	-3.69824
C	-14.8018	-0.01531	-4.12379
C	-15.17243	-1.36798	-4.18488
S	-13.87177	-2.37766	-3.49619
H	-13.04974	1.18556	-3.62195
H	-15.46981	0.77838	-4.39228
C	12.98124	0.19604	2.32363
C	13.78998	-0.87467	2.72911
C	15.00934	-0.42236	3.28782
C	15.12932	1.00211	3.28736
S	13.89043	1.71173	2.27291
H	13.50967	-1.90814	2.64668
H	15.76103	-1.09048	3.66807
C	16.21824	1.75432	4.08011
C	17.98285	3.45819	4.47134
C	17.82205	5.0311	2.6677
C	16.69079	3.0106	3.82672
H	16.67602	1.23524	4.88336
O	18.40976	3.01868	5.57298
S	16.1577	4.30294	2.75582
C	18.06217	6.04678	1.77869
N	18.6964	4.38867	3.58256
C	20.22193	4.39404	3.70024
H	20.48323	3.51511	4.25528
H	20.63271	4.36224	2.72056
C	17.08653	6.37023	0.79414
C	19.25244	6.82497	1.80516
N	16.27534	6.61698	0.00084
N	20.18949	7.47899	1.78213
C	20.85613	5.62135	4.40323
H	21.93617	5.48646	4.41705
H	20.61827	6.50062	3.86137
H	20.51002	5.71078	5.41202
C	-16.43753	-1.69356	-4.75088
C	-18.39319	-2.98362	-5.64349
C	-17.87255	-5.29847	-5.4659
C	-17.05209	-2.90044	-4.91105
H	-16.9943	-0.86816	-5.12112
O	-19.11588	-1.97828	-5.8498
S	-16.59423	-4.52283	-4.45926
C	-17.91104	-6.65806	-5.49055
N	-18.70542	-4.35326	-6.12088
C	-19.74257	-4.50236	-7.25163
H	-19.88857	-3.51978	-7.66723
H	-19.3571	-5.16885	-7.97525
C	-16.79216	-7.34901	-4.93801
C	-18.99208	-7.40958	-5.97966
N	-15.88744	-7.90771	-4.49123
N	-19.85795	-8.06311	-6.37021
C	-21.13827	-5.05798	-6.82724
H	-21.76668	-5.1266	-7.71175

H	-21.01466	-6.02603	-6.40753
H	-21.61625	-4.41489	-6.11565
